# Additional pathways of sterol metabolism: Evidence from analysis of *Cyp27a1−/−* mouse brain and plasma

**DOI:** 10.1016/j.bbalip.2018.11.006

**Published:** 2019-02

**Authors:** William J. Griffiths, Peter J. Crick, Anna Meljon, Spyridon Theofilopoulos, Jonas Abdel-Khalik, Eylan Yutuc, Josie E. Parker, Diane E. Kelly, Steven L. Kelly, Ernest Arenas, Yuqin Wang

**Affiliations:** a Swansea University Medical School, ILS1 Building, Singleton Park, Swansea SA2 8PP, UK; b Laboratory of Molecular Neurobiology, Department of Medical Biochemistry and Biophysics, Karolinska Institutet, Stockholm SE-17177, Sweden

**Keywords:** CYP27A1, Cerebrotendinous xanthomatosis, Oxysterol, Cholestenoic acid, Brain, Mass spectrometry

## Abstract

Cytochrome P450 (CYP) 27A1 is a key enzyme in both the acidic and neutral pathways of bile acid biosynthesis accepting cholesterol and ring-hydroxylated sterols as substrates introducing a (25R)26-hydroxy and ultimately a (25R)26-acid group to the sterol side-chain. In human, mutations in the *CYP27A1* gene are the cause of the autosomal recessive disease cerebrotendinous xanthomatosis (CTX). Surprisingly, *Cyp27a1* knockout mice (*Cyp27a1−/−*) do not present a CTX phenotype despite generating a similar global pattern of sterols. Using liquid chromatography – mass spectrometry and exploiting a charge-tagging approach for oxysterol analysis we identified over 50 cholesterol metabolites and precursors in the brain and circulation of *Cyp27a1−/−* mice. Notably, we identified (25R)26,7α- and (25S)26,7α-dihydroxy epimers of oxysterols and cholestenoic acids, indicating the presence of an *additional* sterol 26-hydroxylase in mouse. Importantly, our analysis also revealed elevated levels of 7α-hydroxycholest-4-en-3-one, which we found increased the number of oculomotor neurons in primary mouse brain cultures. 7α-Hydroxycholest-4-en-3-one is a ligand for the pregnane X receptor (PXR), activation of which is known to up-regulate the expression of CYP3A11, which we confirm has sterol 26-hydroxylase activity. This can explain the formation of (25R)26,7α- and (25S)26,7α-dihydroxy epimers of oxysterols and cholestenoic acids; the acid with the former stereochemistry is a liver X receptor (LXR) ligand that increases the number of oculomotor neurons in primary brain cultures. We hereby suggest that a lack of a motor neuron phenotype in some CTX patients and *Cyp27a1−/−* mice may involve increased levels of 7α-hydroxycholest-4-en-3-one and activation PXR, as well as increased levels of sterol 26-hydroxylase and the production of neuroprotective sterols capable of activating LXR.

## Highlights

• Besides CYP27A1 an additional sterol 26-hydroxylase is present in mouse.
• Sterol-acids are observed as 7α-hydroxy-(25R/S)26-acid epimers.
• The (25S)26-acid is found in mouse brain of the CYP27A1−/− mouse.
• The (25R)26-acid is found in brain of the wild type animal.
• Both epimers are found in plasma of both genotypes.

## Graphical abstract

**Figure:**
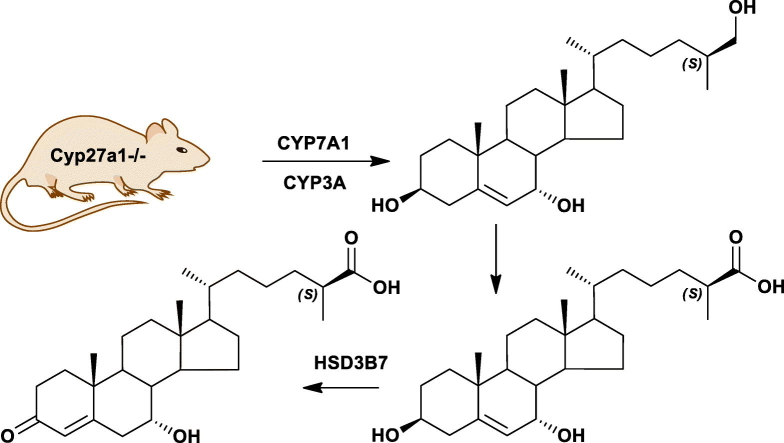

## 1 Introduction

**Fig. 1:**
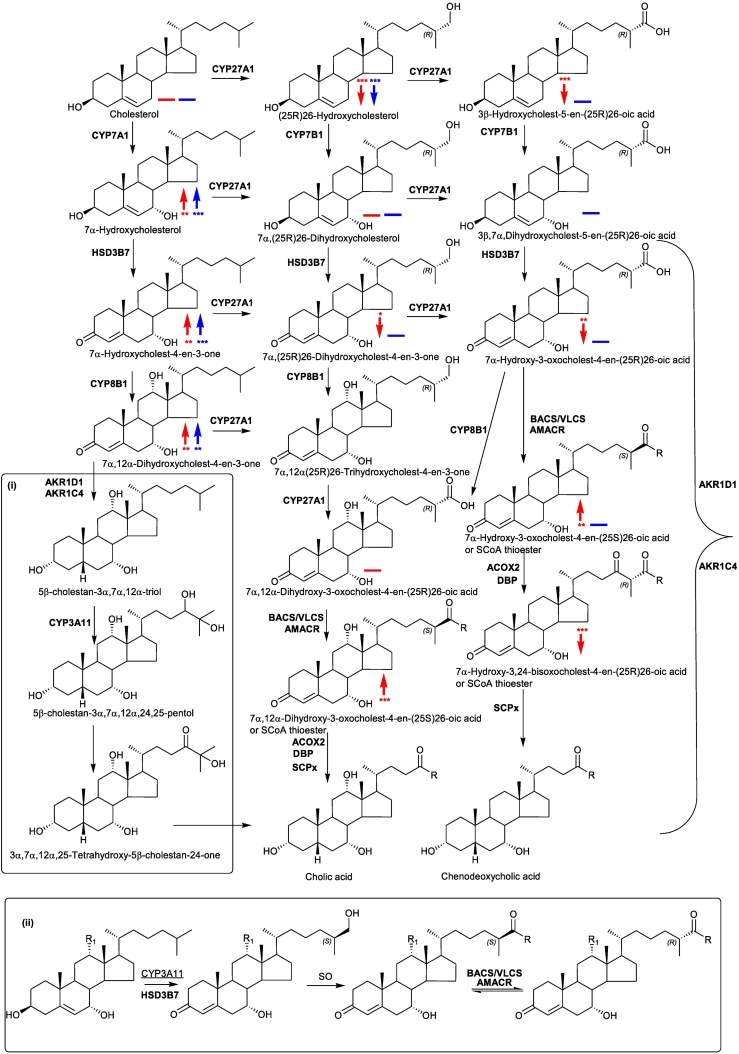
Bile acid biosynthesis via the neutral, acidic, 25-hydroxylase and (25S)26-hydroxylase pathways. The 25-hydroxylase pathway is shown in inset (i), the mouse (25S)26-hydroxylase pathway in inset (ii). R = OH in acids or SCoA in CoA thioesters. R_1_ = H or OH. Where known, mouse enzymes are indicated in **bold**, CYP3A11 was found in the present work to introduce a (25S)26-hydroxy group to 7α-hydroxycholesterol as indicated by underlining of the enzyme symbol. The enzyme which converts the (25S)26-primary alcohol to a carboxylic acid is indicated as a sterol oxidase (SO). Abbreviations: CYP, cytochrome P450; HSD, hydroxysteroid dehydrogenase; AKR, aldo-keto reductase; BACS, bile acyl-CoA synthetase (SLC27A5); VLCS, very long chain acyl-CoA synthetase (SLC27A2); AMACR, alpha-methylacyl-CoA racemase; ACOX2, branched chain acyl-CoA oxidase 2, also called branched-chain acyl-CoA oxidase; DBP, D-bifunctional protein or multifunctional enzyme type 2 (HSD17B4); SCPx, sterol carrier protein x. [3,4]. Metabolites of increased or decreased abundance in the *Cyp27a1−/−* mouse are indicated by upward or downward arrows. Red arrows are used to indicate changes in plasma, blue arrows for brain. A solid horizontal line indicates detected but not significantly changed. *, *P* < 0.05; ** *P* < 0.01; *** *P* < 0.001. *P* < 0.05 is considered significant. The low levels of di- and tri-hydroxycholesterols and of dihydroxycholestenoic acids in brain makes it difficult to distinguish between these compounds and their 3-oxo equivalents using EADSA as their differentiation is based on peak area *difference* between samples treated *with* and *without* cholesterol oxidase (see Fig. S1). Hence, for these metabolites the combined values for the two structures are used.

Cerebrotendinous xanthomatosis (CTX) is an autosomal recessive disease caused by a defective sterol (25R)26-hydroxylase enzyme, also known as sterol 27-hydroxylase, (cytochrome P450 27A1, CYP27A1) [1,2]. In early infancy it can present with cholestatic liver disease, in early childhood with chronic diarrhoea and cataracts, in later childhood with tendon xanthomata, learning difficulties or psychiatric illness and in adult life with spastic paraparesis, a fall in IQ or frank dementia, ataxia and/or dysarthia [1]. Patients with CTX often present with premature atherosclerosis. CYP27A1 is the first enzyme in the acidic pathway of bile acid biosynthesis, it oxidises the terminal carbon of the cholesterol *iso*octyl side-chain first to an alcohol and subsequently to an acid introducing R stereochemistry at C-25 [3,4] (Fig. 1). The resulting products are (25R)26-hydroxycholesterol (cholest-5-ene-3β,(25R)26-diol) and 3β-hydroxycholest-5-en-(25R)26-oic acid, respectively. Note, here we adopt the systematic nomenclature [5] recommended by the Lipid Maps consortium [6], although in much of the literature the non-systematic names 27-hydroxycholesterol and cholestenoic acid are adopted for these two products of CYP27A1 oxidation of cholesterol. CYP27A1 is also an essential enzyme of the neutral pathway of bile acid biosynthesis oxidising ring hydroxylated sterols at C-26 ultimately to 25R-acids. In light of its importance in bile acid biosynthesis, it is not surprising that sterol (25R)26-hydroxylase deficiency leads to disease in humans. Interestingly, although the formation of chenodeoxycholic acid (CDCA, 3α,7α-dihydroxy-5β-cholan-24-oic acid) is greatly reduced in CTX patients, biosynthesis of the other primary bile acid, cholic acid (3α,7α,12α-trihydroxy-5β-cholan-24-oic acid), is maintained [7]. This is achieved via the cholic acid precursor 3α,7α,12α,25-tetrahydroxy-5β-cholestan-24-one and elimination of the terminal three carbons as acetone with the formation of cholic acid (see Fig. 1 inset i) [8]. CTX can be treated by bile acid replacement therapy, especially with CDCA [9] and in combination with low–density lipoprotein (LDL)–apheresis and/or statins [[10], [11], [12]]. Surprisingly, knock-out of the *Cyp27a1* gene in mouse (i.e. *Cyp27a1−/−* mouse) does not lead to a CTX-like phenotype, although production of bile acids is markedly reduced [13].

In both human and mouse, CYP27A1 is expressed ubiquitously. Human macrophages utilise CYP27A1 to generate 3β-hydroxycholest-5-en-(25R)26-oic acid [14], offering a route for “cholesterol transport” back to the liver. In human 7α,(25R)26-dihydroxycholest-4-en-3-one and 7α-hydroxy-3-oxocholest-4-en-(25R)26-oic acid are biosynthesised in brain, either from cholesterol or from imported (25R)26-hydroxycholesterol, utilising CYP27A1, CYP7B1 and subsequently hydroxysteroid dehydrogenase (HSD) 3B7, and are exported from brain into the circulation providing another route for “cholesterol transport” to liver [[15], [16], [17]]. Interestingly, both 3β-hydroxycholest-5-en-(25R)26-oic acid and 3β,7α-dihydroxycholest-5-en-(25R)26-oic acid, precursors of 7α-hydroxy-3-oxocholest-4-en-(25R)26-oic acid, are ligands to the liver X receptors (LXRs) in neuronal cells, but the 3-oxo acid is not [18,19]. 7α,(25R)26-Dihydroxycholesterol (cholest-5-ene-3β,7α,(25R)26-triol), a precursor of 7α,(25R)26-dihydroxycholest-4-en-3-one, and its positional isomer 7α,25-dihydroxycholesterol (cholest-5-ene-3β,7α,25-triol), have activity in the immune system as ligands to the G protein-coupled receptor (GPCR), Epstein-Barr virus induced gene 2 (GPR183) [20,21], while 7β,(25R)26-dihydroxycholesterol (cholest-5-ene-3β,7β,(25R)26-triol) has been reported to be a ligand to the nuclear receptor RORγ (RAR-related orphan receptor gamma) [22], and (25R)26-hydroxy-7-oxocholesterol (3β,(25R)26-dihydroxycholest-5-en-7-one) and its isomer 25-hydroxy-7-oxocholesterol (3β,25-dihydroxycholest-5-en-7-one) activate the hedgehog signalling pathway by binding to the GPCR Smoothened [23].

Considering the importance of CYP27A1 in bile acid biosynthesis in human, and the biological activity of intermediates in the acidic pathway of bile acid synthesis [[18], [19], [20], [21]], it is intriguing that the *Cyp27a1−/−* mice do not show a disease phenotype. In the current study we have investigated the profile of metabolites (>50 sterols, oxysterols and sterol-acids) involved in the bile acid biosynthetic pathways in the *Cyp27a1−/−* mouse, concentrating on the circulation and the brain. Our results show that the *Cyp27a1−/−* mouse synthesises both oxysterols and cholestenoic acids with a 25S-stereochemistry. As the 25R- and 25S-epimers of the acids are inter-convertible this provides an *additional* route to the synthesis of 3β,7α-dihydroxycholest-5-en-(25R)26-oic acid and also to bile acids. We have shown earlier that 3β,7α-dihydroxycholest-5-en-(25R)26-oic acid promotes the survival of motor neurons [19]. Patients with CTX, but not *Cyp27a1−/−* mice, may present with motor neuron dysfunction, this difference could be explained by the presence of an *additional* route to synthesis of the neuroprotective acid in mouse. We hereby show the loss of *Cyp27a1* in mice increases the levels of 7α-hydroxycholest-4-en-3-one, a pregnane X receptor (PXR) ligand known to increase the levels of CYP3A11 [24], resulting in expression of a murine sterol 26-hydroxylase and a pathway to neuroprotective LXR ligands.

## 2 Materials and methods

### 2.1 Oxysterol analysis

We adopted a charge-tagging approach utilising “enzyme-assisted derivatisation for sterol analysis” (EADSA) to enhance liquid chromatography (LC) separation and mass spectrometry (MS) detection of oxysterols [17,25,26]. This involves the stereospecific enzymatic oxidation of the 3β-hydroxy-5-ene function in oxysterols (and sterols) to a 3-oxo-4-ene group and subsequent reaction with the cationic Girard P (GP) hydrazine reagent to give charged GP-hydrazones compatible with chromatographic separation using reversed phase solvents and high-sensitivity analysis by electrospray ionisation (ESI)-MS and MS with multistage fragmentation (MS^n^) (Fig. S1). GP-derivatives give intense [M]^+^ ions in ESI and informative MS^2^ and MS^3^ spectra. As some oxysterols naturally contain a oxo group they give GP-derivatives even in the absence of oxidising enzyme. However, oxysterols containing a *native* 3-oxo group are readily differentiated from oxysterols *oxidised* to contain one by dividing each sample in two and performing derivatisation *without* oxidising enzyme on one portion of the sample (Fraction B) and performing derivatisation *with* added enzyme on the second portion (Fraction A), and by exploiting differentially isotope labelled GP reagents to allow discrimination by mass (Fig. S1).

Unless otherwise indicated, materials and methods are as described by Griffiths and co-workers [17,25,26]. Quantification was achieved using isotope dilution mass spectrometry. No hydrolysis or solvolysis steps were performed. 3β,7α-Dihydroxycholest-5-en-(25S)26-oic acid was supplied as a mixture with 3β,7α-dihydroxycholest-5-en-(25R)26-oic acid (1:3, mole:mole) as the [3α,7β-^2^H_2_] compounds by Avanti Polar Lipids (Alabaster, AL, USA).

### 2.2 CYP3A4 and CYP3A11 incubations

Recombinant mouse CYP3A11 in bactosomes (Cypex Ltd., Dundee, UK) or recombinant human 3A4 in baculosomes (Life Technologies now Thermo Fisher Scientific) (10 pmol) was incubated with 1–10 μg of 7α-hydroxycholesterol (cholest-5-ene-3β,7α-diol), 2 mM NADPH, 5 mM glucose-6-phosphate and 0.4 U glucose-6-dehydrogenase in 0.1 M potassium phosphate buffer, pH 7.4, at a final volume of 500 μL for 16 h at 37 °C. The reaction was quenched with ethyl acetate and the organic phase dried down for oxysterol analysis as above. Negative control experiments were performed in the absence of enzyme or NADPH. Incubations with CYP125, a known 26-hydroxylase, were performed to provide a positive control.

### 2.3 Animals

Male mouse plasma and brain samples were purchased from The Jackson Laboratory (USA). *Cyp27a1−/−* mice (*Cyp27a1*^*tm1Elt*^, *n* = 3, 3 months of age) had no RNA or protein expression from *Cyp27a1* in liver tissue [13]. Control mice (*Cyp27a1+/+*, n = 3, 3 months of age) were C57BL/6J strain from the same colony.

### 2.4 Luciferase reporter assay

The ability of oxysterols and various cholesterol metabolites to activate nuclear receptors i.e. PXR, constitutive androstane receptor (CAR), LXR, farnesoid X receptor (FXR), vitamin D receptor (VDR) and nuclear receptor related protein 1 (NURR1) was tested in luciferase assays. Transient transfections were performed in the mouse substantia nigra–like cell line SN4741. Cells were plated in 24-well plates (5 × 10^5^ cells per well) 24 h before transfection, transfected with 1 μg of plasmid DNA per well and complexed with 2 μL of Lipofectamine 2000 (Invitrogen). Cells were transfected with 400 ng of a PXR-, CAR-, LXR-, FXR-, VDR-, or NURR1-responsive luciferase reporter construct and 200 ng PXR, CAR, LXRα, FXR, VDR, or NURR1 [19,24,27]. A reporter gene expressing the Renilla luciferase (pRL-TK, Promega) was co-transfected in all experiments as an internal control for normalization of transfection efficiency. After a 12 h incubation, the lipid/DNA mix was replaced with fresh 2.5% serum medium containing vehicle or appropriate ligand (10 μM), as specified in each experiment. Luciferase activities were assayed 24 h later using the Dual-Luciferase Reporter Assay System (Promega), following the manufacturer's protocol.

### 2.5 Primary midbrain cultures

Brains from E11.5 mouse embryos were obtained, the midbrain region was dissected, mechanically dissociated and plated on poly-d-lysine (150,000 cells/cm^2^) and grown in serum-free N2 media consisting of 1:1 mixture of F12 and DMEM with 10 ng/mL insulin, 100 μg/mL apo-transferrin, 100 μM putrescine, 20 nM progesterone, 30 nM selenium, 6 mg/mL glucose and 1 mg/mL BSA. Cells were treated for 3 days in vitro (DIV) with the compounds of interest, fixed with 4% PFA and processed for staining using appropriate antibodies. The fixed cells were washed in PBS and blocked in 5% normal goat serum/PBS for 1 h at room temperature. Primary antibodies were diluted in PBS (pH 7.4), 0.3% Triton X-100, 1% BSA and incubations were carried out overnight at +4 °C or at room temperature for 2 h. The antibodies used were anti-: Islet-1 (1:100; Developmental Studies Hybridoma Bank) and Nkx6.1 (1:200; Novus Biologicals) and appropriate secondary antibodies (Jackson ImmunoResearch or Alexa). Cells positive for the corresponding marker were counted directly at the microscope at a magnification of 20×. Cells were counted in every well, in eight consecutive fields (going from one side of the well to the other, passing through the center), in three different wells per experiment and in three different experiments per condition. Random pictures of the wells were taken for every condition to document the result, and representative pictures were subsequently selected to represent the quantitative data. Photos were acquired with a Zeiss Axioplan microscope and a Hamamatsu camera C4742–95 using the Openlab software.

### 2.6 Statistics

For oxysterol and sterol analysis data are means ± standard deviation (SD), *, *P* < 0.05; **, *P* < 0.01; ***, *P* < 0.001 by Student's *t*-test. For nuclear receptor luciferase assays data are means ± standard error of the mean (SEM), *, *P* < 0.05; **, *P* < 0.01 by Mann-Whitney test, compared to vehicle treatment. For the quantification of Islet-1+ cells, *, *P* < 0.05 by Mann-Whitney test, compared to vehicle treatment.

### 2.7 Ethical approval

Ethical approval for mice experimentation was granted by Stockholm Norra Djurförsöksetisks Nämnd number N154/06, N145/09, N370/09 and N273/11.

## 3 Results and discussion

### 3.1 Analysis of plasma cholesterol and metabolite levels in the Cyp27a1−/− mouse

#### 3.1.1 Monohydroxycholesterols and monohydroxycholestenones

**Fig. 2:**
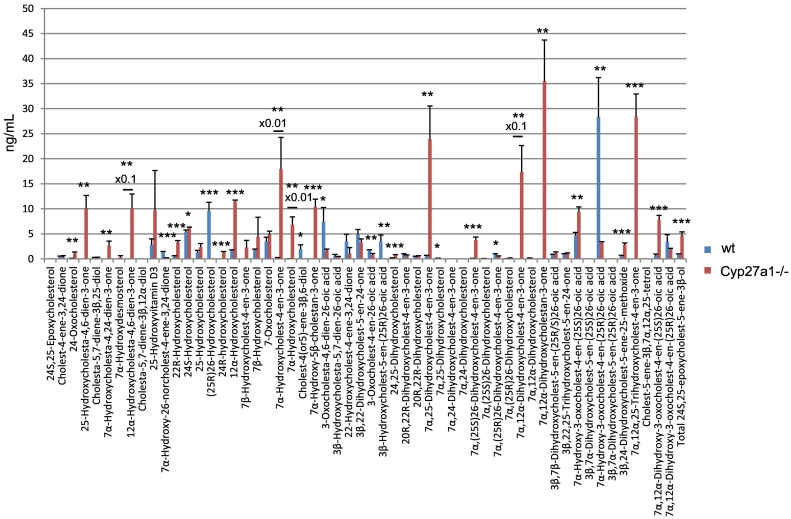
Concentrations of oxysterols and cholestenoic acids in *Cyp27a1−/−* (*n* = 3) and *Cyp27a1+/+* (wt, n = 3) mouse plasma. No hydrolysis or solvolysis steps were performed so the values reported are for “free” non-esterified molecules. Sterols are arranged according to mass and chromatographic order of elution of the GP-derivative. To maintain a single y-axis magnification factors have been applied as indicated. Using the EADSA method 24S,25-epoxycholesterol isomerises to 24-oxocholesterol, becomes hydrolysed to 24,25-dihydroxycholesterol and undergoes methanolysis to 3β,24-dihydroxycholest-5-ene-25-methoxide. The total 24S,25-epoxycholesterol corresponds to the sum of the individual forms.

**Fig. 3:**
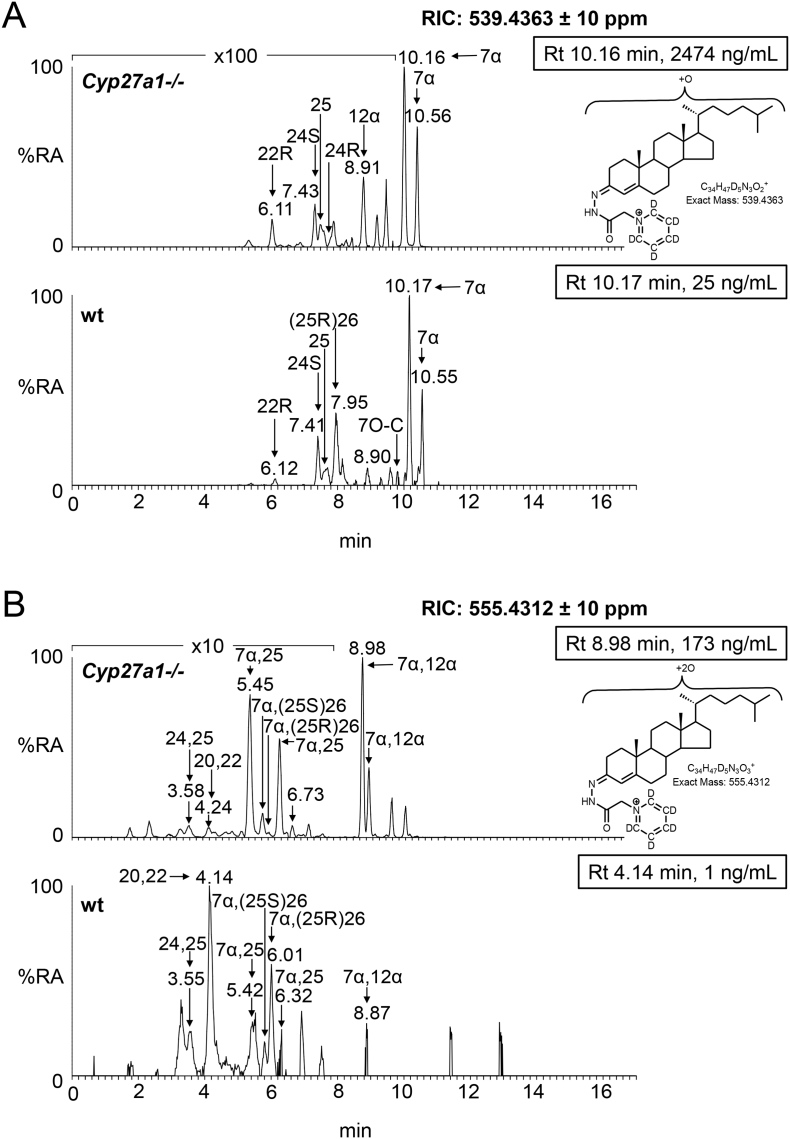

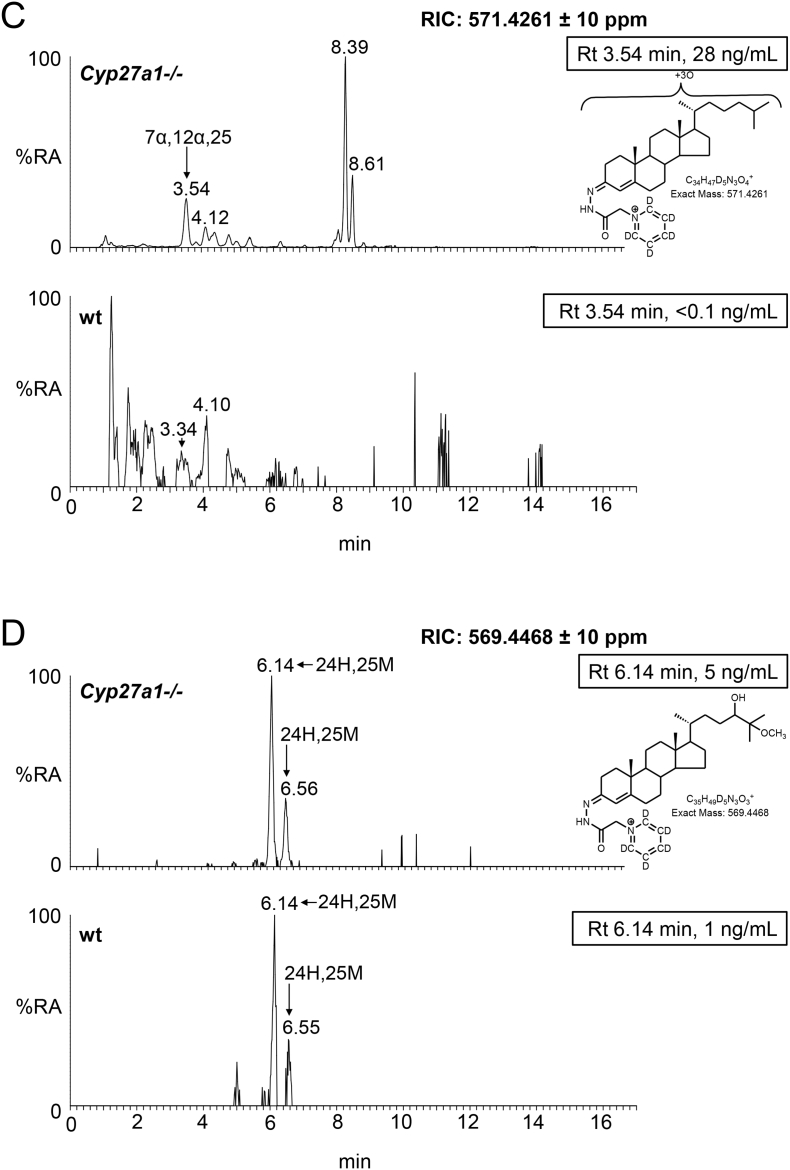
Oxysterols in *Cyp27a1−/−* and *Cyp27a1+/+* (wt) mouse plasma. Each chromatogram is normalised to the most intense peak at 100% relative abundance (RA). Magnification factors are as indicated. The concentration of the indicated analyte (by retention time, Rt) is given in the right-hand corner of each chromatogram. Chromatograms from the oxysterol fractions treated with cholesterol oxidase (combination of sterols with a *native* 3-oxo group and those *oxidised* by cholesterol oxidase to contain a 3-oxo group) are shown. The insets show structures of generic GP derivatives. Many oxysterols elute as twin peaks corresponding to *syn* and *anti* conformers. (A) Monohydroxycholesterols and monohydroxycholest-4-en-3-ones. (B) Dihydroxycholesterols and dihydroxycholest-4-en-3-ones. (C) Trihydroxycholesterols and trihydroxycholest-4-en-3-ones. (D) 3β,24-Dihydroxycholest-5-ene-25-methoxide, the methanolysis product of 24S,25-epoxycholesterol. In (A-C) peaks are labelled with the location of the relevant hydroxy groups on the cholesterol or cholest-4-en-3-one structure. In (D) 3β,24-Dihydroxycholest-5-ene-25-methoxide is abbreviated to 24H,25 M.

While the dominant oxysterol in both genotypes is 7α-hydroxycholest-4-en-3-one its level varies dramatically from 20.68 ± 6.04 ng/mL (mean ± SD) in the wild type (wt) to 1792.87 ± 634.79 ng/mL in the *Cyp27a1−/−* animals (Fig. 2, Fig. 3A, Table S1). This is explained by an up-regulation of the cholesterol 7α-hydroxylase enzyme (CYP7A1) in the *Cyp27a1−/−* animals as a consequence of reduced negative feedback by primary bile acids on its expression [13]. The immediate product of CYP7A1 catalysed hydroxylation of cholesterol, 7α-hydroxycholesterol, is subsequently oxidised by HSD3B7 to 7α-hydroxycholest-4-en-3-one (Fig. 1) [3]. The level of 7α-hydroxycholesterol is also greatly elevated in *Cyp27a1−/−* mouse plasma (680.78 ± 157.99 ng/mL cf. 4.05 ± 1.96 ng/mL). Sterol 12α-hydroxylase (CYP8B1) is regulated in a similar manner to CYP7A1 in mouse [3] and thus is expected to be up-regulated in the *Cyp27a1−/−* mouse. This may account for an increase in the intensity of the peak at 8.91 min in the reconstructed ion chromatogram (RIC) appropriate for monohydroxycholesterols (Fig. 3A), which we annotate as 12α-hydroxycholesterol (cholest-5-ene-3β,12α-diol, 11.47 ± 0.29 ng/mL cf. 1.62 ± 0.19 ng/mL). Note we use here the term annotate to label a metabolite in the absence of an authentic standard or reference spectra. 12α-Hydroxycholesterol is not commercially available, but has been shown by Danielsson to be converted to cholic acids by rats and rabbits [28]. As expected, a significant difference in the pattern of monohydroxycholesterols is the absence of (25R)26-hydroxycholesterol in *Cyp27a1−/−* mouse plasma (<0.1 ng/mL). This oxysterol is present at a level of about 10 ng/mL in the wt mouse plasma. Similar observations with respect to 7α-hydroxycholesterol and (25R)26-hydroxycholesterol were made by Rosen et al. in serum and by Honda et al. with respect to (25R)26-hydroxycholesterol in liver mitochondria and to 7α-hydroxycholesterol and 7α-hydroxycholest-4-en-3-one in liver microsomes in their studies on *Cyp27a1*−/− animals [13,29]. As determined here, Mast et al. found 7α-hydroxycholest-4-en-3-one to be much more abundant in plasma from *Cyp27a1−/−* mice than wt animals [30]. Levels of brain-derived 24S-hydroxycholesterol (cholest-5-ene-3β,24S-diol) [3] show a small but significant increase in the *Cyp27a1−/−* mouse (6.34 ± 0.01 ng/mL cf. 5.40 ± 0.37 ng/mL) perhaps as a consequence of reduced metabolism [31], as do levels of 22R-hydroxycholesterol (cholest-5-ene-3β,22R-diol, 3.28 ± 0.41 ng/mL cf. 0.60 ± 0.02 ng/mL), the intermediate formed during side-chain shortening in steroid hormone biosynthesis by the enzyme CYP11A1. 25-Hydroxycholesterol (cholest-5-ene-3β,25-diol) did not vary between genotypes (<2.5 ng/mL). This is also true of 7-oxocholesterol (3β-hydroxycholest-5-en-7-one, <5 ng/mL). An interesting observation is the presence of 24R-hydroxycholesterol (cholest-5-ene-3β,24R-diol) in plasma of the *Cyp27a1−/−* mouse (1.28 ± 0.19 ng/mL), this elutes very close to (25R)26-hydroxycholesterol, and its low level is usually obscured by the latter oxysterol which is typically about an order of magnitude more abundant in wt animals [32,33].

#### 3.1.2 Dihydroxycholesterols and dihydroxycholestenones

In the wt animal, only low levels (<1 ng/mL) of these oxysterols are observed, the most abundant being 20R,22R-dihydroxycholesterol (cholest-5-ene-3β,20R,22R-triol), 20R,22R-dihydroxycholest-4-en-3-one, 7α,25-dihydroxycholest-4-en-3-one and 7α,(25R)26-dihydroxycholest-4-en-3-one (Fig. 2, Fig. 3B, Table S1). The situation in the *Cyp27a1−/−* mouse is very different in that high levels of 7α,12α-dihydroxycholest-4-en-3-one (173.11 ± 53.45 ng/mL) and appreciable levels of 7α,25-dihydroxycholest-4-en-3-one (23.86 ± 6.68 ng/mL) are observed. These differences between genotypes are not surprising, as both CYP7A1 and 8B1 are up-regulated in the *Cyp27a1−/−* mouse as is the microsomal 25-hydroxylase CYP3A11 [34]. 7α,25-Dihydroxycholest-4-en-3-one may be formed via 25-hydroxycholesterol and subsequently 7α,25-dihydroxycholesterol, or alternatively via 7α-hydroxycholesterol followed by 25-hydroxylation of either 7α-hydroxycholesterol or 7α-hydroxycholest-4-en-3-one (Fig. S2). Cholesterol 25-hydroxylase (CH25H) is the enzyme responsible for formation of the majority of 25-hydroxycholesterol and levels of this oxysterol do not differ significantly between wt and *Cyp27a1−/−* mice, however, the activity of CYP3A11, an alternative sterol 25-hydroxylase, is up-regulated in the *Cyp27a1−/−* mouse [34], and could account for the hydroxylation at C-25 of 7α-hydroxycholesterol or 7α-hydroxycholest-4-en-3-one to ultimately give 7α,25-dihydroxycholest-4-en-3-one. Microsomal CYP3A11 has in addition to 25-hydroxylase activity, 23R-, 24R-, 24S- and 26-hydroxylase activities [34]. In our in vitro incubation of recombinant mouse CYP3A11 with 7α-hydroxycholesterol we observed 25- and (25S)26-hydroxylation (see section 3.3.). Inspection of the RICs appropriate to dihydroxycholesterols and dihydroxycholestenones clearly reveals 7α,(25R)26-dihydroxycholest-4-en-3-one (0.86 ± 0.19 ng/mL) in wt mouse plasma. In contrast, its epimer, 7α,(25S)26-dihydroxycholest-4-en-3-one (3.79 ± 0.56 ng/mL) is predominant in the *Cyp27a1−/−* mouse. Although we do not have an authentic synthetic standard of 7α,(25S)26-dihydroxycholest-4-en-3-one its identical MS^3^ spectrum to that of 7α,(25R)26-dihydroxycholest-4-en-3-one and slightly earlier elution on reversed phase LC is entirely compatible with our annotation. This slight shift towards early elution is seen with other oxysterols with 25S-stereochemistry compared to their 25R-epimers [35,36]. Only trace levels of 7α,(25S)26-dihydroxycholest-4-en-3-one (<0.1 ng/mL) are found in plasma from the wt mice and low levels of 7α,(25R)26-dihydroxycholest-4-en-3-one (<0.5 ng/mL) in plasma from the *Cyp27a1−/−* mouse.

#### 3.1.3 Trihydroxycholesterols and trihydroxycholestenones

In light of the elevated abundance of both 7α,12α-dihydroxycholest-4-en-3-one and of 7α,25-dihydroxycholest-4-en-3-one in plasma from the *Cyp27a1−/−* mouse, it might be expected that their down-stream metabolite 7α,12α,25-trihydroxycholest-4-en-3-one would also be elevated in plasma. As predicted, in the appropriate RIC (Fig. 3C) a cholesterol metabolite rises in concentration from about the detection limit (0.05 ng/mL) in the wt mice to 28.40 ± 4.52 ng/mL in the *Cyp27a1−/−* animals (Fig. 2, Table S1). Although no authentic standard was available the MS^3^ spectrum is entirely compatible with the proposed 7α,12α,25-trihydroxycholest-4-en-3-one structure. It is noteworthy that Honda et al. identified the down-stream metabolite 5β-cholestane-3α,7α,12α,25-tetrol in liver microsomes from *Cyp27a1−/−* animals [29].

#### 3.1.4 Hydroxycholestenoic acids

**Fig. 4:**
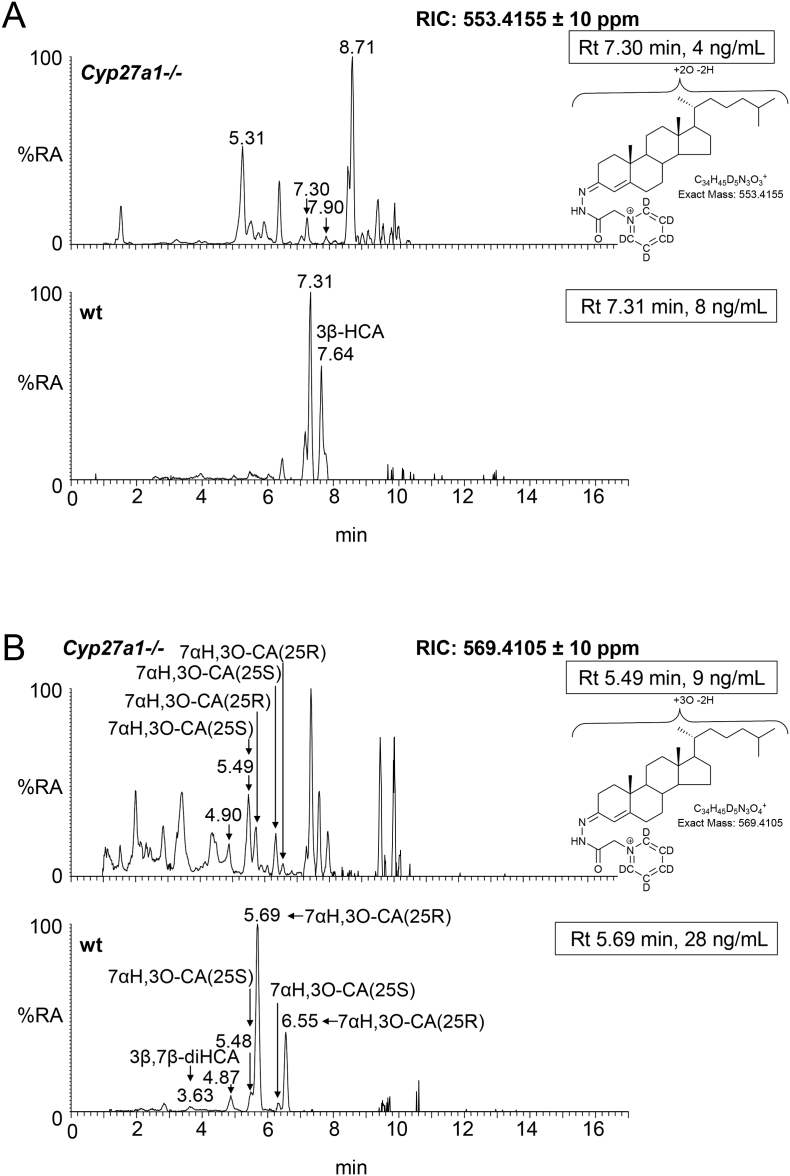

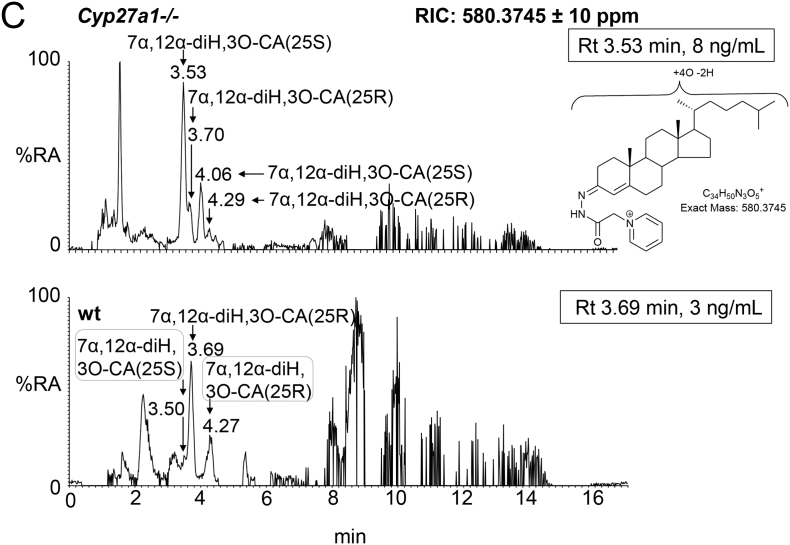
Cholestenoic acids in *Cyp27a1−/−* and *Cyp27a1+/+* (wt) mouse plasma. Each chromatogram is normalised to the most intense peak at 100% RA. Magnification factors are as indicated. The concentration of the indicated analyte (by Rt) is given in the right-hand corner of each chromatogram. Chromatograms from the oxysterol fractions treated with cholesterol oxidase (combination of sterols with a *native* 3-oxo group and those *oxidised* by cholesterol oxidase to contain a 3-oxo group) are shown in (A)–(B). The insets show structures of generic GP derivatives. Many cholestenoic acids elute as twin peaks corresponding to *syn* and *anti* conformers. (A) 3β-Hydroxychest-5-enoic and 3-oxocholest-4-enoic acids, and dihydroxycholestenones and hydroxycholestenediones. (B) Dihydroxycholestenoic and hydroxyoxocholestenoic acids. (C) Dihydroxyoxocholestenoic acids. Abbreviations: 3β-HCA, 3β-hydroxycholest-5-en-(25R)26-oic; 7αH,3O-CA(25R or S), 7α-hydroxy-3-oxocholest-4-en-(25R or S)26-oic; 3β,7β-diHCA, 3β,7β-dihydroxycholest-5-en-26-oic; and 7α,12α-diH,3O-CA(25R or S), 7α,12α-dihydroxy-3-oxocholest-4-en-(25R or S)26-oic acids. The peaks at 7.30 min and 7.31 min in (A) are annotated to a combination of 3β,22-dihydroxycholest-5-en-24-one and 22-hydroxycholest-4-ene-3,24-dione. The peaks at 4.90 min and 4.87 min in (B) are annotated to 3β,22,25-trihydroxycholest-5-en-24-one.

3β-Hydroxycholest-5-en-(25R)26-oic acid is present in appreciable amounts in plasma from the wt animals (3.56 ± 1.22 ng/mL) but is essentially absent from plasma of the *Cyp27a1−/−* animals (Fig. 2, Fig. 4A, Table S1). This result is in agreement with the lack of detectable levels of its precursor (25R)26-hydroxycholesterol in plasma from *Cyp27a1−/−* animals. No evidence for the 25S-epimer of the acid was found in plasma of either genotype.

#### 3.1.5 Dihydroxycholestenoic and hydroxyoxocholestenoic acids

In the wt animals we see appreciable levels of 7α-hydroxy-3-oxocholest-4-en-(25R)26-oic acid (28.36 ± 7.87 ng/mL). We also see evidence for the presence of its epimer 7α-hydroxy-3-oxocholest-4-en-(25S)26-oic acid but at much lower levels (4.63 ± 0.65 ng/mL, Fig. 2, Fig. 4B, Table S1). In the *Cyp27a1−/−* mouse we see the opposite situation with 7α-hydroxy-3-oxocholest-4-en-(25S)26-oic acid (9.34 ± 1.04 ng/mL) being about three times as abundant as 7α-hydroxy-3-oxocholest-4-en-(25R)26-oic acid (3.23 ± 0.23 ng/mL). This data indicates that there is a second mechanism in mouse to oxidise 7α-hydroxycholest-4-en-3-one and/or 7α-hydroxycholesterol to cholestenoic acids, independent of CYP27A1 (Fig. 1, see inset ii). Once formed the 25S- and 25R-acids can isomerise in reactions catalysed by α-methylacyl-CoA racemase (AMACR) following activation to their CoA-thioesters [3,4,37]. In both the wt and *Cyp27a1−/−* mice low levels of 3β,7β-dihydroxycholest-5-en-(25R/S)26-oic acid (about 1 ng/mL) are observed. HSD3B7 is inactive towards 7β-hydroxy substrates, requiring a 7α-hydroxy group in the substrate, hence the 3-oxo-4-ene product is not observed.

In wt mouse 7α-hydroxy-3-oxocholest-4-en-(25R)26-oic acid undergoes A-ring reduction and side-chain shortening through a complex series of reactions leading to the formation of CDCA (Fig. 1). Cytosolic aldo-keto reductases (AKR) 1D1 and 1C4 catalyse A-ring reduction, while side-chain shortening of the CoA thioester proceeds in the peroxisome catalysed by AMACR, branched chain acyl-CoA oxidase 2 (ACOX2), D-bifundtional protein (DBP, HSD17B4) and sterol carrier protein x (SCPx) [3]. The substrate for the final step of this series is the CoA thioester of the 24-oxo-(25R)26-carboxylic acid. With our analytical method we tend to observe the carboxylic acids rather than their CoA thioesters and β-keto acids are unstable and decompose by loss of CO_2_ to the 26-*nor*-24-ketones [18]. As might be predicted, the concentration of 7α-hydroxy-26-*nor*-cholest-4-ene-3,24-dione is reduced from 1.33 ± 0.14 ng/mL in the wt mouse to 0.18 ± 0.05 ng/mL in the *Cyp27a1−/−* mouse. We do not have an authentic standard for 7α-hydroxy-26-*nor*-cholest-4-ene-3,24-dione, but retention time and MS^3^ spectrum are compatible with the proposed structure.

#### 3.1.6 Trihydroxycholestenoic and dihydroxyoxocholestenoic acids

The RICs appropriate to trihydroxycholestenoic and dihydroxyoxocholestenoic acids are shown in Fig. 4C. Each of the four labelled peaks gives essentially an identical MS^3^ spectrum. Considering wt plasma first, we assign the peaks at 3.69 and 4.27 min to the *syn* and *anti* forms of GP-derivatised 7α,12α-dihydroxy-3-oxocholest-4-en-(25R)26-oic acid (3.39 ± 1.46 ng/mL). In plasma from the *Cyp27a1−/−* mice the intensities of these two peaks are reduced (1.94 ± 0.18 ng/mL), although not significantly, however, two earlier eluting peaks at 3.53 and 4.06 min are enhanced which we assign to the *syn* and *anti* forms of the 7α,12α-dihydroxy-3-oxocholest-4-en-(25S)26-oic acid (7.79 ± 0.88 ng/mL). These peaks are only minor in plasma from the wt animals (0.63 ± 0.31 ng/mL). The identification of the 25R- and 25S-epimers was confirmed in studies of the AMACR knockout mouse [32]. In combination, this data reinforces the notion that there is an *additional* route to introducing a carboxylic acid group to the terminal carbon of the sterol *iso*octyl side-chain in mouse besides that provided CYP27A1 (Fig. 1, insert ii). Honda et al. have proposed that mouse hepatic CYP3A11 has 26-hydroxylase activity towards 5β-cholestane-3α,7α,12α-triol and 5β-cholestane-3α,7α,12α,25-tetrol and that the expression of this enzyme is up-regulated in the *Cyp27a1−/−* mouse [34]. Our data suggest that this, and/or another enzyme, also oxidises 7α,12α-dihydroxycholest-4-en-3-one at the C-26 position to the (25S)26-sterol acid. Once formed the (25S)26- and (25R)26-sterol acids are inter-convertible [37].

#### 3.1.7 24S,25-Epoxycholesterol

Unlike other oxysterols, 24S,25-epoxycholesterol (3β-hydroxycholest-5-en-24S,25-epoxide) is formed through a shunt of the mevalonate pathway, specifically the Bloch arm of the pathway (Fig. S3). In the shunt pathway squalene epoxidase (SQLE) introduces two oxygen atoms into squalene rather than one and the enzyme 24-dehydrocholesterol reductase (DHCR24) is not involved. We observe low levels of 24S,25-epoxycholesterol (<1 ng/mL) in plasma of the wt animal, but significantly higher amounts in plasma of the *Cyp27a1−/−* animals (4.66 ± 0.72 ng/mL, Fig. 2, Fig. 3D, Table S1). Rosen et al. found that hepatic levels of HMG-CoA reductase (*Hmgcr*) mRNA are 2–3 fold higher in the *Cyp27a1−/−* animals than wt [13], while Båvner et al. found elevated levels of lathosterol, a marker of cholesterol synthesis, in liver of *Cyp27a1−/−* mice [38]. Thus, our data supports the concept of an enhanced flow of metabolites through the mevalonate pathway in *Cyp27a1−/−* mice and an increase in cholesterol biosynthesis. An alternative route to 24S,25-epoxycholesterol has recently been suggested by Goyal et al., who show that the human cholesterol 24S-hydroxylase (CYP46A1) enzyme can oxidise desmosterol (cholesta-5,24-dien-3β-ol) to the 24S,25-epoxide (Fig. S3) [39]. An elevation of 24S,25-epoxycholesterol formed via this route would similarly be compatible with an increase in flow of metabolites through the mevalonate pathway. An alternative explanation could be that knockout of *Cyp27a1* removes a route for epoxide metabolism.

#### 3.1.8 Other oxysterols

A number of other oxysterols were found which differ in abundance between the two mouse genotypes. While 7α-hydroxydesmosterol (cholesta-5,24-diene-3β,7α-diol, 0.29 ± 0.34 ng/mL) and its down-stream metabolite 7α-hydroxycholesta-4,24-dien-3-one (2.59 ± 0.95 ng/mL) are presumptively identified (by retention time and MS^3^ spectra in the absence of authentic standards) in plasma from the *Cyp27a1−/−* mice, they are absent from the wt plasma (<0.1 ng/mL) (Fig. 2, Fig. S4A, Table S1). This can be explained by the up-regulated CYP7A1 enzyme using desmosterol as a substrate in the *Cyp27a1−/−* mouse (Fig. S2, inset i). In plasma from the *Cyp27a1−/−* mouse, at least two other metabolites with the same mass as 7α-hydroxycholesta-4,24-dien-3-one are observed, but are absent in the wt plasma. Based on MS^3^ spectra, these are annotated as 25-hydroxycholesta-4,6-dien-3-one (10.03 ± 2.63 ng/mL) and 12α-hydroxycholesta-4,6-dien-3-one (101.14 ± 28.42 ng/mL). Båvner et al. have proposed the formation of cholesta-4,6-dien-3-one from 7α-hydroxycholest-4-en-3-one in *Cyp27a1−/−* mice (Fig. S2, inset ii) [38]. The diene may then act as a substrate for up-regulated CYP8B1 or CYP3A11 to introduce hydroxy groups at C-12α or C-25, respectively. Alternatively, the microsomal enzyme, which dehydrates 7α-hydroxycholest-4-en-3-one to cholesta-4,6-dien-3-one [40] may similarly dehydrate 7α,12α-dihydroxycholest-4-en-3-one and 7α,25-dihydroxycholest-4-en-3-one to the respective 4,6-dienes. 7α-Hydroxycholest-4-en-3-one is also a substrate for Δ^4^–3-oxosteroid 5β-reductase (AKR1D1), which reduces the 4–5-double bond giving 7α-hydroxy-5β-cholestan-3-one [3]. When a RIC is plotted for the appropriate *m/z* from *Cyp27a1−/−* plasma (Fig. S4B) a peak is observed which we annotate to 7α-hydroxy-5β-cholestan-3-one (10.35 ± 1.59 ng/mL, Fig. 2, Table S1). This peak is absent in analysis of wt plasma. DeBarber et al., also using a LC-MS method with derivatisation, similarly found a metabolite of elevated abundance in *Cyp27a1−/−* mouse plasma, which was annotated to 7α-hydroxy-5β-cholestan-3-one [41]. The enzyme AKR1D1 will similarly reduce 7α,12α-dihydroxycholest-4-en-3-one and probably 7α,25-dihydroxycholest-4-en-3-one to their 5β-cholestan-3-one equivalents. The RIC of the appropriate *m/z* 552.4160 reveals two peaks (Fig. S4C), one of which has a retention time compatible with 7α,12α-dihydroxy-5β-cholestane-3-one (35.49 ± 8.20 ng/mL). This peak is absent when wt plasma is analysed.

#### 3.1.9 25-Hydroxyvitamin D_3_

In their initial study, Rosen et al. using a radioimmunoassay found that *Cyp27a1−/−* mice had somewhat higher serum concentrations of 25-hydroxyvitamin D_3_ (9,10-*seco*cholesta-5,7,10(19)-triene-3β,25-diol) than wt animals [13]. In the present study we similarly find an elevated level of this *seco*sterol in *Cyp27a1−/−* mouse plasma (9.66 ± 7.98 ng/mL cf. 2.80 ± 1.17 ng/mL) but the difference with wt is not significant (Fig. 2, Fig. S4A, Table S1).

#### 3.1.10 Cholesterol, desmosterol, 7(or8)-dehydrocholesterol, 7(or8)-dehydrodesmosterol, cholestenone and cholestanol

The concentration of non-esterified cholesterol in plasma of the *Cyp27a1−/−* mouse (242.79 ± 28.39 μg/mL) is similar to that of the wt (223.22 ± 7.23 μg/mL, Figs. S5A, S6A, Table S1). Rosen et al. also found this to be true for total cholesterol [13], although Båvner et al. found a small but significant fall in cholesterol levels in the male *Cyp27a1−/−* mouse [38]. Surprisingly, we find a significant reduction in desmosterol levels in the *Cyp27a1−/−* mouse compared to the wt (0.14 ± 0.03 μg/mL cf. 0.28 ± 0.05 μg/mL). This would argue against an up-regulation of the Bloch arm of the mevalonate pathway suggested above (section 3.1.7) to explain the increase in 24S,25-epoxycholesterol in plasma from the *Cyp27a1−/−* mouse. However, an alternative explanation for a fall in desmosterol levels is an increase in its metabolism in the *Cyp27a1−/−* mouse, i.e. through 7α-hydroxylation by up-regulated CYP7A1. Båvner et al. found increased lathosterol levels in liver of the *Cyp27a1−/−* mouse indicating up-regulation of the Kandutsch-Russell arm of the mevalonate pathway [38]. This is supported by our finding of elevated levels of 8(9)-dehydrocholesterol (cholesta-5,8(9)-dien-3β-ol) in the *Cyp27a1−/−* mouse (0.64 ± 0.21 μg/mL cf. 0.07 ± 0.09 μg/mL). 8(9)-Dehydrocholesterol is an enzymatically formed isomer of 7-dehydrocholesterol (cholesta-5,7-dien-3β-ol, Fig. S3) [42]. Two other sterols detected in plasma were cholesta-4,6-dien-3-one and cholest-4-en-3-one. Neither showed a significant change in concentration in plasma for the two genotypes, although an increase in cholesta-4,6-dien-3-one in the *Cyp27a1−/−* animal tended towards significance (0.21 ± 0.05 μg/mL cf. 0.07 ± 0.08 μg/mL, *P* = 0.06). Likewise, DeBarber et al. also found higher levels of cholesta-4,6-dien-3-one in plasma of the *Cyp27a1−/−* mouse than in the wt [41]. A final metabolite detected in both *Cyp27a1−/−* and wt mice was a cholestatrien-3β-ol. The MS^3^ spectrum suggests that this corresponds to 7-dehydrodesmosterol (cholesta-5,7,24-trien-3β-ol) or its Δ8 isomer. This metabolite is more abundant in *Cyp27a1−/−* plasma than in the wt (0.06 ± 0.02 μg/mL cf. 0.02 ± 0.01 μg/mL). 7-Dehydrodesmosterol is the immediate precursor of desmosterol in the Bloch pathway. We were unable to detect cholestanol (5α-cholestan-3β-ol) in plasma from either mouse genotypes. In studies by others, cholestanol has been found to be elevated in the *Cyp27a1−/−* mouse [30]. Our inability to detect cholestanol in this study is probably a dynamic range issue as a consequence of its low abundance in comparison to cholesterol which elutes close by in our LC system.

### 3.2 Analysis of brain cholesterol and metabolite levels in the Cyp27a1−/− mouse

As humans with CTX can show spastic paraparesis, a fall in IQ or frank dementia and ataxia we next examined the oxysterol and sterol profile of brain from *Cyp27a1−/−* mice and wt controls.

#### 3.2.1 Monohydroxycholesterols and monohydroxycholestenones

**Fig. 5:**
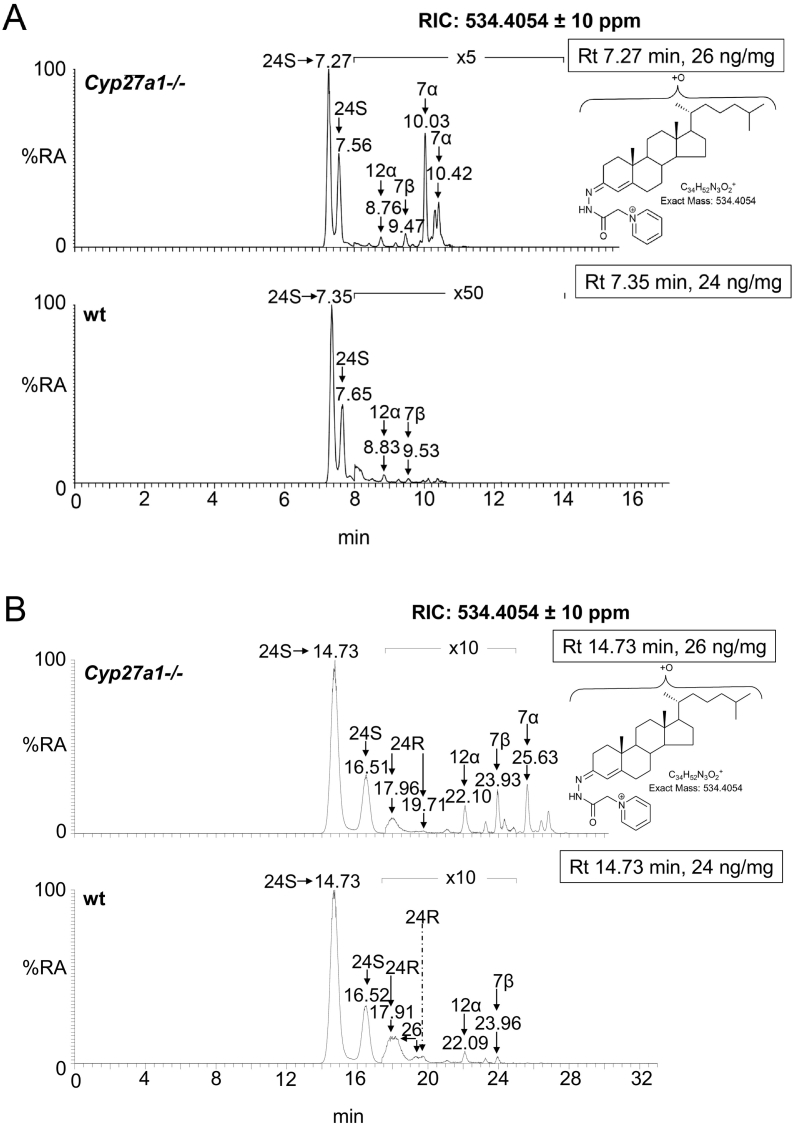

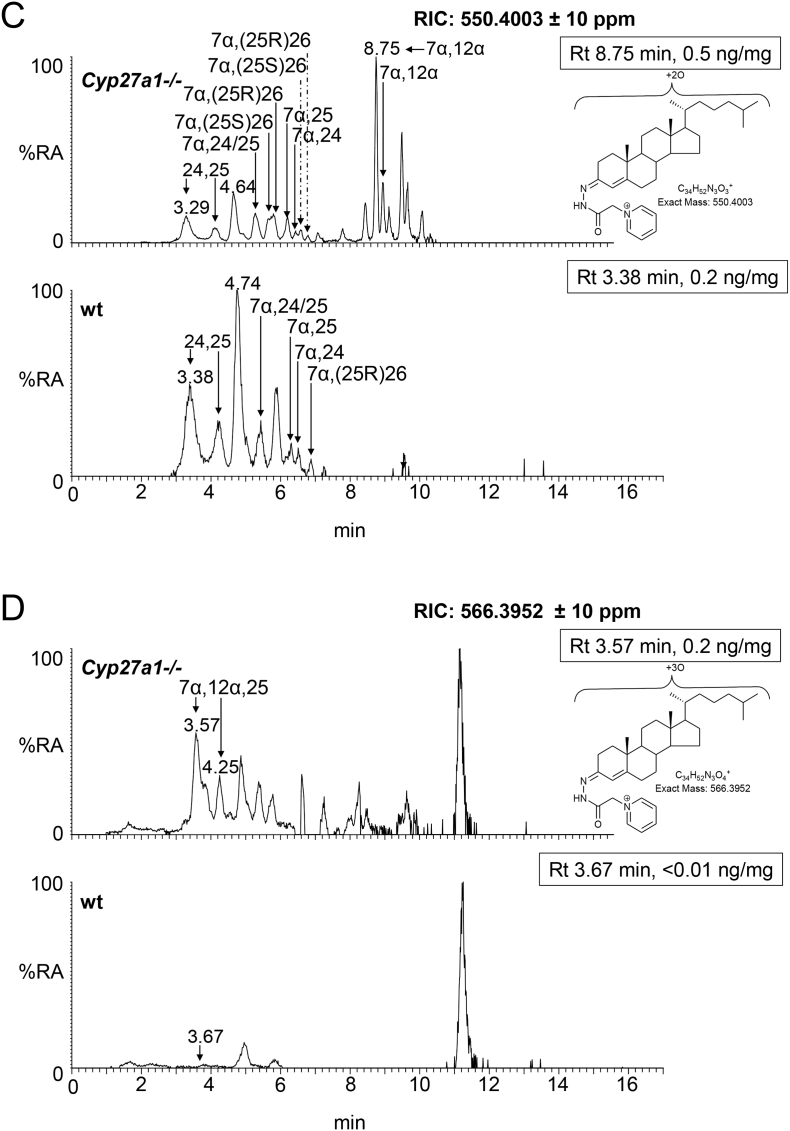

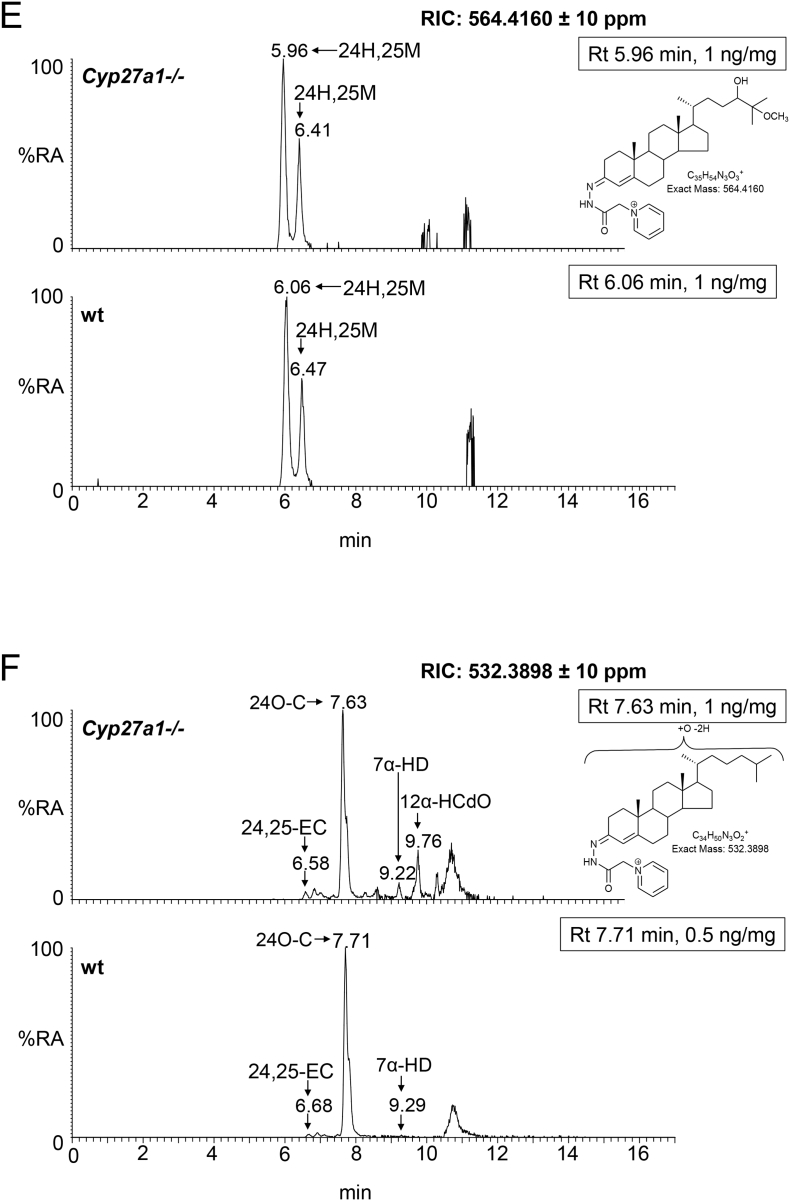
Oxysterols in *Cyp27a1−/−* and *Cyp27a1+/+* (wt) mouse brain. Each chromatogram is normalised to the most intense peak at 100% RA. Magnification factors are as indicated. The concentration of the indicated analyte (by Rt) is given in the right-hand corner of each chromatogram. Chromatograms from the oxysterol fractions treated with cholesterol oxidase (combination of sterols with a *native* 3-oxo group and those *oxidised* by cholesterol oxidase to contain a 3-oxo group) are shown. The insets show structures of generic GP derivatives. Many oxysterols elute as twin peaks corresponding to *syn* and *anti* conformers. (A) Monohydroxycholesterols and monohydroxycholest-4-en-3-ones. (B) As in (A) but over an extended gradient. (C) Dihydroxycholesterols and dihydroxycholest-4-en-3-ones. (D) Trihydroxycholesterols and trihydroxycholest-4-en-3-ones. (E) 3β,24-Dihydroxycholest-5-ene-25-methoxide, the methanolysis product of 24S,25-epoxycholesterol. (F) Monohydroxycholestenones, monohydroxydehydrocholesterols and monohydroxycholesta-4,6(or24)-dien-3-ones. In (A-D) peaks are labelled according to the respective location of hydroxy groups on the core cholesterol or cholest-4-en-3-one structure. In (E) and (F) the abbreviations are 24H,25 M, 3β,24-dihydroxycholest-5-ene-25-methoxide; 24,25-EC, 24S,25-epoxycholesterol; 24O-C, 3β-hydroxycholest-5-en-24-one; 7α-HD, 7α-hydroxydesmosterol; 12α-HCdO, 12α-hydroxycholestadien-3-one.

**Fig. 6:**
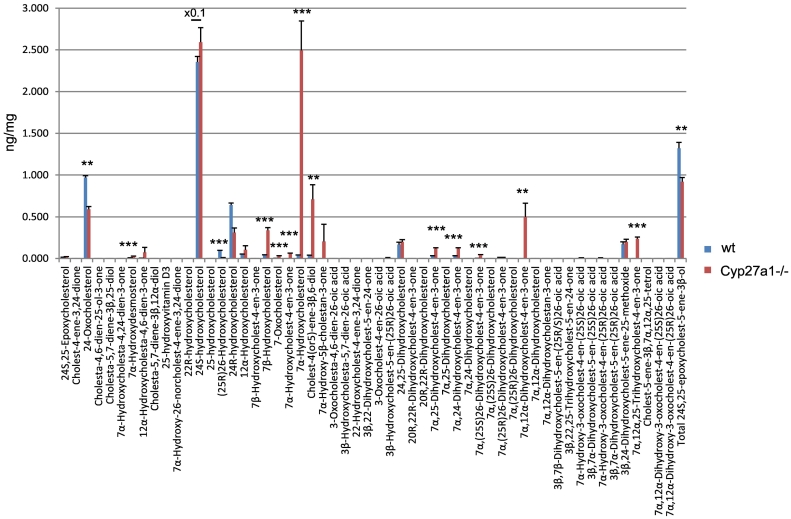
Concentrations of oxysterols and cholestenoic acids in *Cyp27a1−/−* (*n* = 3) and *Cyp27a1+/+* (wt, n = 3) mouse brain. No hydrolysis or solvolysis steps were performed so the values reported are for “free” non-esterified molecules. Sterols are arranged according to mass and chromatographic order of elution of the GP-derivative. To maintain a single y-axis magnification factors have been applied as indicated. The low levels of di- and tri-hydroxycholesterols and of dihydroxycholestenoic acids in brain made it difficult to distinguish between these compounds and their 3-oxo equivalents using EADSA as their differentiation is based on peak area *difference* between samples treated *with* and *without* cholesterol oxidase. Hence, for these metabolites the combined values for the two structures are given, and for simplicity we just give values for di- and trihydroxycholest-4-en-3-ones and 7α-hydroxy-3-oxocholest-4-enoic acids. Using the EADSA method 24S,25-epoxycholesterol isomerises to 24-oxocholesterol, becomes hydrolysed to 24,25-dihydroxycholesterol and undergoes methanolysis to 3β,24-dihydroxycholest-5-ene-25-methoxide. The total 24S,25-epoxycholesterol corresponds to the sum of the individual forms.

The dominant oxysterol in both *Cyp27a1−/−* and wt mouse brain is 24S-hydroxycholesterol and the levels are approximately equal at about 25 ng/mg (Figs. 5A & B, 6, Fig. S7 Table S1). Similarly, Ali et al. also found approximately equal levels of 24S-hydroxycholesterol in brain cortex of the two genotypes [43], however, Mast et al. found lower levels of 24S-hydroxycholesterol in whole brain and in cerebellum of the *Cyp27a1−/−* mouse than wt [30]. While here, and in the study by Ali et al., measurements are in ng/mg, Mast et al. used the units pmol/mg protein [30,43]. Low levels of the 24R-epimer are also observed in each genotype (<1 ng/mg). Only trace amounts of (25R)26-hydroxycholesterol are observed in the wt mice (≤0.1 ng/mg), but as expected this oxysterol is essentially absent in the *Cyp27a1−/−* mice (≤0.01 ng/mg). It is difficult to resolve 24R-hydroxycholesterol from (25R)26-hydroxycholesterol even using an extended gradient (Fig. 5B) [35]. In contrast to (25R)26-hydroxycholesterol, there is an increase in the level of 7α-hydroxycholesterol in the brain of *Cyp27a1−/−* mice compared to controls (2.49 ± 0.35 ng/mg cf. 0.04 ± 0.01 ng/mg). This is also seen for 7α-hydroxycholest-4-en-3-one (0.06 ± 0.01 ng/mg cf. <0.01 ng/mg), 7-oxocholesterol (0.03 ± 0.00 ng/mg cf. 0.00 ± 0.00 ng/mg), 7β-hydroxycholesterol (cholest-5-ene-3β,7β-diol, 0.34 ± 0.03 ng/mg cf. 0.04 ± 0.01 ng/mg) and 6β-hydroxycholesterol (cholest-4-ene-3β,6β-diol, 0.71 ± 0.17 ng/mg cf. 0.04 ± 0.00 ng/mg). 6β-Hydroxycholesterol is formed in our derivatisation procedure by hydrolysis of cholestane-3β,5α,6β-triol, a metabolite formed from both 5α- and 5β-epimers of 5,6-epoxycholesterol (3β-hydroxycholestan-5,6-epoxide) in a reaction catalysed by cholesterol epoxide hydrolase [44]. Cholestane-3β,5α,6β-triol is known to be converted to the bile acid 3β,5α,6β-trihydroxycholanoic acid in a pathway involving CYP27A1 [[45], [46], [47]]. The absence of CYP27A1 in brain of the *Cyp27a1−/−* mouse may explain the elevation of the cholestane-3β,5α,6β-triol surrogate, 6β-hydroxycholesterol, observed here. As CYP7A1, the enzyme that generates 7α-hydroxycholesterol, is not reportedly expressed in brain [3], it is likely that the origin of 7α-hydroxycholesterol and 7α-hydroxycholest-4-en-3-one is extracerebral. In a previous study of the *Cyp27a1−/−* mouse, Rosen et al. also found elevated levels of 7α-hydroxycholesterol in brain [13] and Båvner et al. suggested that this oxysterol crosses the blood brain barrier (BBB) from the circulation to brain [38]. The parallel increase in abundance of 7-oxocholesterol and 7β-hydroxycholesterol with 7α-hydroxycholesterol and 7α-hydroxycholest-4-en-3-one in brain suggests that the former two oxysterols may be related to the latter two in terms of biosynthesis and/or metabolism. This concept is supported by recent publications by Shinkyo et al. [48] and Björkhem et al. [49] who both showed that human CYP7A1 can convert 7-dehydrocholesterol to 7-oxocholesterol, while Larssen et al. showed that 7-oxocholesterol and 7β-hydroxycholesterol are inter-convertible in man [50], the enzyme catalysing this reaction in man and in mouse being 11β-hydroxysteroid dehydrogenase type 1 (HSD11B1) [51]. All four 7-oxidised forms of cholesterol are known to be metabolised by CYP27A1 and the absence of this enzyme in *Cyp27a1−/−* mouse brain is the likely driver to their increase seen here [47]. As in plasma, we observe a metabolite which we annotate as 12α-hydroxycholesterol in brain of the *Cyp27a1−/−* mouse, but unlike in plasma its level does not differ significantly between the two genotypes (0.10 ± 0.05 ng/mg cf. 0.05 ± 0.01 ng/mg).

#### 3.2.2 Dihydroxycholesterols and dihydroxycholestenones

The low levels of these metabolites made it difficult to distinguish between dihydroxycholesterols and dihydroxycholestenones, as using EADSA their differentiation is based on peak area *difference* between samples treated *with* and *without* cholesterol oxidase. Hence, for these metabolites the combined values for the two structures are given, and for simplicity we just refer to them as dihydroxycholestenones. The pattern of dihydroxycholestenones in brain of both the *Cyp27a1−/−* and wt mice is complex (Fig. 5C). In the *Cyp27a1−/−* and wt mice we observe both 7α,25- and 7α,24-dihydroxycholest-4-en-3-ones. In the *Cyp27a1−/−* animals we see an elevation in the levels of these metabolites compared to wt. As the first peaks of the *syn*/*anti* pairs of both oxysterols almost co-elute we measured the combined amount for the two oxysterols (Figs. 5C, 6, Table S1). This gave a value of 0.12 ± 0.01 ng/mg in the *Cyp27a1−/−* animals compared with 0.03 ± 0.00 ng/mg in the wt. As in plasma, we find 7α,(25S)26-dihydroxycholest-4-en-3-one in brain of the *Cyp 27a1−/−* mouse (0.04 ± 0.00 ng/mg) and also traces of the 25R epimer (0.01 ± 0.00 ng/mg). In the wt animal the 25R epimer is present at trace levels (0.01 ± 0.00 ng/mg) while the 25S isomer is essentially absent (<0.01 ng/mg). The exact origin of these 7α,26-dihydroxy metabolites in brain is not clear; a possible origin in the *Cyp27a1−/−* mice is that they formed in brain by side-chain hydroxylation of imported 7α-hydroxycholesterol or 7α-hydroxycholest-4-en-3-one by up-regulated CYP3A11 (Fig. 1, inset ii), which is reported to be expressed in brain [52]. Alternatively, CYP46A1, which is abundantly expressed in brain has 25-hydroxylase, (25R)26-hydroxylase as well as 24S-hydroxylase activity to cholesterol and can also use 7α-hydroxycholesterol as a substrate and thus may account for some of the dihydroxy metabolites found in the two genotypes (Fig. S7) [53]. There is likely to be also some direct 7α-hydroxylation of side-chain hydroxylated substrates by CYP7B1 accounting for 7α,(25R)26-dihydroxy metabolites in the wt brain. CYP7B1 is an oxysterol 7α-hydroxylase which is expressed in brain and 7α-hydroxylates both 25- and (25R)26-hydroxycholesterols but has only minor activity towards 24S-hydroxycholesterol [3,54], however, this latter substrate is greatly dominating in brain, and its hydroxylation by CYP7B1 may account for the observation of some of the 7α-hydroxy metabolite. CYP39A1, the oxysterol 7α-hydroxylase which acts on 24S-hydroxycholesterol is expressed in many tissues. The *Cyp39a1* gene is reported to be expressed in the somatosensory cortex of adult mouse brain [55]. The major dihydroxycholestenone in *Cyp27a1−/−* brain is 7α,12α-dihydroxycholest-4-en-3-one, this oxysterol is not detected in wt brain (0.50 ± 0.17 ng/mg cf. <0.01 ng/mg). Its high level in plasma suggests it may be imported from the circulation into brain.

#### 3.2.3 Trihydroxycholesterols and trihydroxycholestenones

As above, combined values for these two generic structures were recorded, and for simplicity we just refer to them as trihydroxycholestenones. As in plasma from the *Cyp27a1−/−* mice we see a trihydroxycholestenone in brain, which we annotate with the structure 7α,12α,25-trihydroxycholest-4-en-3-one (0.24 ± 0.02 ng/mg, Fig. 5D). There is no evidence for this molecule in wt brain (Fig. 6, Table S1).

#### 3.2.4 Cholestenoic acids

**Fig. 7:**
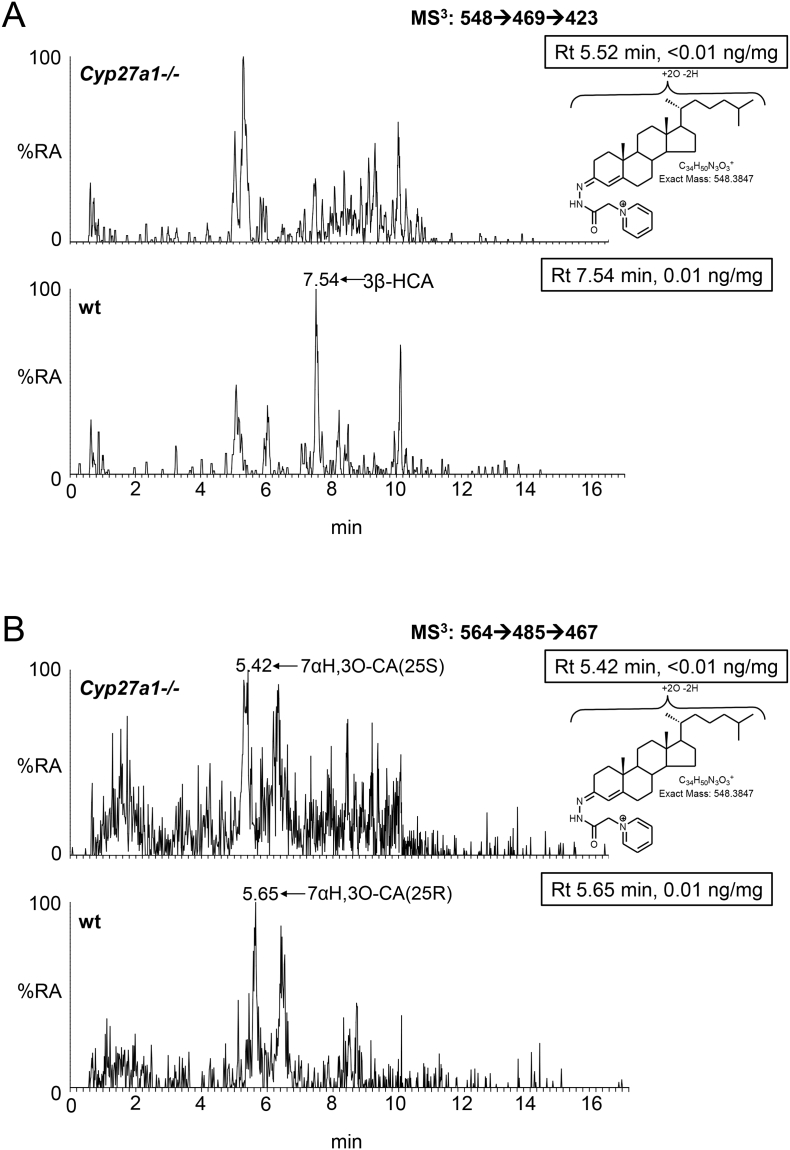
Cholestenoic acids in *Cyp27a1−/−* and *Cyp27a1+/+* (wt) mouse brain. Each chromatogram is normalised to the most intense peak at 100% RA. Magnification factors are as indicated. The concentration of the indicated analyte (by Rt) is given in the right-hand corner of each chromatogram. Chromatograms from the oxysterol fractions treated with cholesterol oxidase (combination of sterols with a *native* 3-oxo group and those *oxidised* by cholesterol oxidase to contain a 3-oxo group) are shown. The insets show structures of generic GP derivatives. Many cholestenoic acids elute as twin peaks corresponding to *syn* and *anti* conformers. (A) 3β-Hydroxychest-5-enoic and 3-oxocholest-4-enoic acids, and dihydroxycholestenones and hydroxycholestenediones. (B) Dihydroxycholestenoic and hydroxyoxocholestenoic acids. Abbreviations: 3β-HCA, 3β-hydroxycholest-5-en-(25R)26-oic; 7αH,3O-CA(25R or S), 7α-hydroxy-3-oxocholest-4-en-(25R or S)26-oic.

As reported earlier only trace levels of 3β-hydroxycholest-5-en-(25R)26-oic and 7α-hydroxy-3-oxocholest-4-en-(25R)26-oic acid are observed in the wt animals (about 0.01 ng/mg, Fig. 6, Fig. 7A & B, Table S1) [19], while only 7α-hydroxy-3-oxocholest-4-en-(25S)26-oic is observed in brain of the *Cyp27a1−/−* animals at the limit of detection (0.01 ng/mg). As above, the values for 7α-hydroxy-3-oxocholest-4-en-(25R)26-oic and its 25S-epimer also include a contribution from the 3β,7α-dihydroxy-acids.

#### 3.2.5 24S,25-Epoxycholesterol

We have reported earlier that the level of 24S,25-epoxycholesterol is reduced in brain of *Cyp27a1−/−* mice (0.92 ± 0.05 ng/mg, cf. 1.32 ± 0.07 ng/mg, Figs. 5E, 6, Table S1) [56]. This is in contrast to the situation in plasma, but is compatible with reduced cholesterol biosynthesis through the Bloch arm of the cholesterol biosynthesis pathway as observed by Ali et al. who found reduced desmosterol levels in cortex of brain of *Cyp27a1−/−* animals [43]. We also find reduced levels of desmosterol in whole brain of the *Cyp27a1−/−* mouse (see section 3.2.7). The current data also supports the recent report of Goyal et al. that shows that 24S,25-epoxycholesterol can be formed from desmosterol in a reaction catalysed by CYP46A1 (Fig. S3) [39].

#### 3.2.6 Other oxysterols

The level of 7α-hydroxydesmosterol is elevated in brain of the *Cyp27a1−/−* mice (0.03 ± 0.00 ng/mg cf. 0.01 ± 0.00 ng/mg, Figs. 5F, 6, Table S1). The oxysterols annotated as 12α-hydroxycholesta-4,6-dien-3-one (0.07 ± 0.06 ng/mg cf. <0.01 ng/mg) and 7α-hydroxy-5β-cholestan-3-one (0.21 ± 0.21 ng/mg cf. <0.01 ng/mg) are also elevated in the Cyp27a1−/− mouse but not to significance.

#### 3.2.7 Cholesterol, desmosterol, 7(or 8)-dehydrocholesterol, cholestenone and cholestadienone

As found by others [30,38,43], and as we reported earlier, the cholesterol levels in brain of the *Cyp27a1−/−* and the wt mouse are essentially the same (16.98 ± 0.96 μg/mg cf. 16.90 ± 0.29 μg/mg, Figs. S6B, S8A, Table S1) [56], while the desmosterol level in brain of the *Cyp27a1−/−* mice is lower than in the wt (0.04 ± 0.00 μg/mg cf. 0.06 ± 0.00 μg/mg), however, the level of 8(9)-dehydrocholesterol is higher in the *Cyp27a1−/−* mice than wt (0.05 ± 0.00 μg/mg cf. 0.01 ± 0.00 μg/mg, Fig. S8C). 8(9)-Dehydrocholesterol is formed from 7-dehydrocholesterol through enzymatic isomerisation (Fig. S3) [42]. This data agrees with that of Båvner et al., Ali et al. and Mast et al. who suggested that the Kandutsch-Russell pathway is up-regulated in brain of the *Cyp27a1−/−* mouse while the Bloch pathway is down-regulated or not changed [30,38,43]. We also observe enhanced amounts of cholest-4-en-3-one (1.30 ± 0.82 μg/mg cf. 0.17 ± 0.06 μg/mg) in brain of the *Cyp27a1−/−* mice compared to wt mice. We did not quantify cholesta-4,6-dien-3-one but it gave a stronger signal in chromatograms from the *Cyp27a1−/−* mouse than the wt. It has been suggested that these two sterols are involved in the formation of cholestanol in CTX. Although we failed to detect cholestanol in our study, probably on account of the limited dynamic range of our LC-MS method and saturating amounts of cholesterol, Båvner et al. and Mast et al. have found elevated levels of cholestanol by GC–MS in *Cyp27a1−/−* mouse brain [30,38]. Cholestanol is suggested to be formed via two mechanisms, one involving dehydrogenation of cholesterol to cholest-4-en-3-one by a 3β-hydroxy-Δ^5^-dehydrogenase (other than HSD3B7) followed by two reductive steps, and the other from 7α-hydroxycholest-4-en-3-one by dehydration followed by saturation of the 6–7 double bond to give cholest-4-en-3-one (Fig. S3, inset i) [57]. The observation of both cholesta-4,6-dien-3-one and cholest-4-en-3-one suggests that the second pathway is utilised in *Cyp27a1−/−* brain, at least to some extent.

### 3.3 Enzyme activity of CYP3A11 and CYP3A4

In a previous study Honda et al. showed that hepatic microsomal 23-, 24-, 25- and 26-hydroxylations of 5β-cholestane-3β,7α,12α-triol and 23R-, 24R-, 24S- and 27-hydroxylations of 5β-cholestane-3β,7α,12α,25-tetrol were catalysed by CYP3A enzymes, CYP3A4 in man and predominantly by CYP3A11 in mouse [34]. Here we performed a preliminary study to investigate whether these two enzymes also had activity towards 7α-hydroxycholesterol, the primary product of CYP7A1 catalysed hydroxylation of cholesterol. We found that recombinant human CYP3A4 hydroxylates 7α-hydroxycholesterol predominantly to 7α,25-dihydroxycholesterol and to a minor extent to 7α,(25S)26-dihydroxycholesterol. Recombinant CYP3A11 generates minor quantities of both 7α,(25S)26-dihydroxycholesterol and 7α,25-dihydroxycholesterol. Reducing the substrate concentration from 50 μM (10 μg/500 μL) to 5 μM reduced the product formation by both enzymes. At 5 μM the concentration of 7α,(25S)26-dihydroxycholesterol formed was below the limit of detection for incubations with either enzyme. In 16 h incubations with 50 μM 7α-hydroxycholesterol, turnover of substrate by CYP3A4 (20 nM) and CYP125 (20 nM), a known 26-hydroxylase, were 3% and 7%, respectively. Negative control experiments in the absence enzyme or NADPH confirmed the requirement of enzyme and co-factor for the formation of both dihydroxycholesterols. In future studies we will investigate the activities of CYP3A enzymes towards other substrates of enhanced abundance in *Cyp27a1−/−* animals. However, this preliminary study confirms mouse CYP3A11 as a (25S)26-hydroxylase to substrates with cholest-5-en-3β,7α-diol structure.

### 3.4 Sterols as nuclear receptor ligands

Sterols, including oxysterols and cholestenoic acids, are known ligands to nuclear receptors, including the LXRs, FXR, PXR, (also known as the steroid xenobiotic receptor, SXR) and VDR [18,19,24,[58], [59], [60], [61]]. Another nuclear receptor, CAR, or constitutive androstane receptor, as the name implies, exhibits an intrinsically high transcriptional activity and provokes activation of target gene expression in the absence of ligand binding, but can also be activated by cholesterol precursors [62]. Of these nuclear receptors, LXRα and β and PXR are known to be expressed in midbrain [63,64], and in previous studies we have identified 24S,25-epoxycholesterol to be a potent LXR ligand that enhances midbrain dopamine neurogenesis in the developing midbrain and 3β,7α-dihydroxycholest-5-en-(25R)26-oic acid, an intermediate in the pathway from cholesterol to bile acids, to be a ligand to the LXRs which enhances motor neuron survival in the CNS via activation of these receptors [19,63].

Some patients with CTX, characterised by deficiency in CYP27A1 and an inability to biosynthesise cholestenoic acids, present with motor neuron disease [65], however, the *Cyp27a1−/−* mouse does not show a motor neuron disease phenotype. In an attempt to explain this anomaly, we have investigated if cholesterol metabolites of enhanced abundance in the *Cyp27a1−/−* mouse are nuclear receptor ligands, and whether they are protective towards motor neurons through activation of nuclear receptors.

**Fig. 8:**
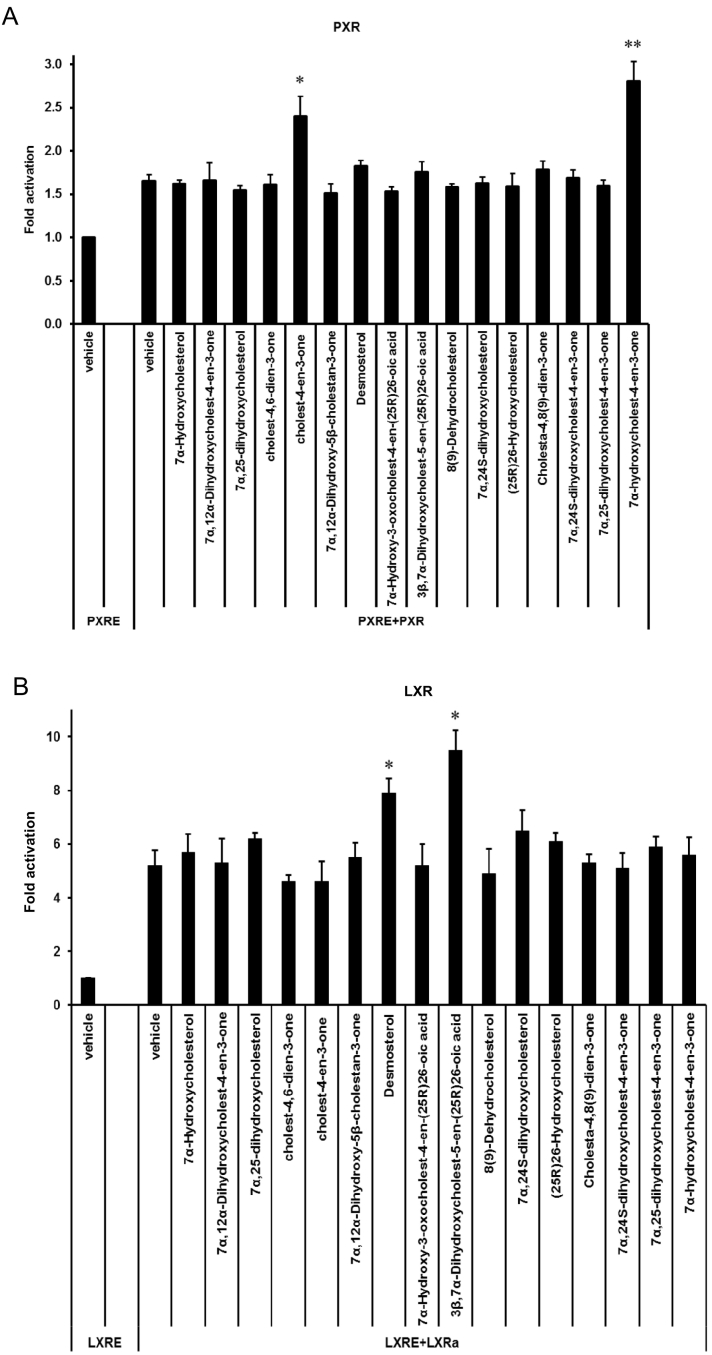
Analysis of the PXR activational capacity of sterols and oxysterols of enhanced or changed abundance in *Cyp27a1−/−* mouse. Luciferase activity in SN4741 neural cells transfected with (A) a PXR-responsive luciferase reporter construct (PXRE) and PXR, and (B) an LXR-responsive luciferase reporter construct (LXRE) and LXRα, and stimulated for 24 h with the compounds indicated (10 μM). Cholest-4-en-3-one and 7α,24-dihydroxycholest-4-en-3-one were increased in brain but not in plasma, 7α,12α-dihydroxycholestan-3-one was elevated in plasma but not in brain, 7α,(25S)26-dihydroxycholest-4-en-3-one and 7α,12α,25-tihydroxycholest-4-en-3-one are not commercially available, while 7α-hydroxy-3-oxocholest-4-en-(25S)26-oic is only available as an unresolved mixture with the 25R epimer. (25R)26-Hydroxycholesterol and desmosterol were reduced in both brain and plasma of the *Cyp27a1−/−* mouse, 7α-hydroxy-3-oxocholest-4-en-(25R)26-oic acid was reduced in plasma only.

**Fig. 9:**
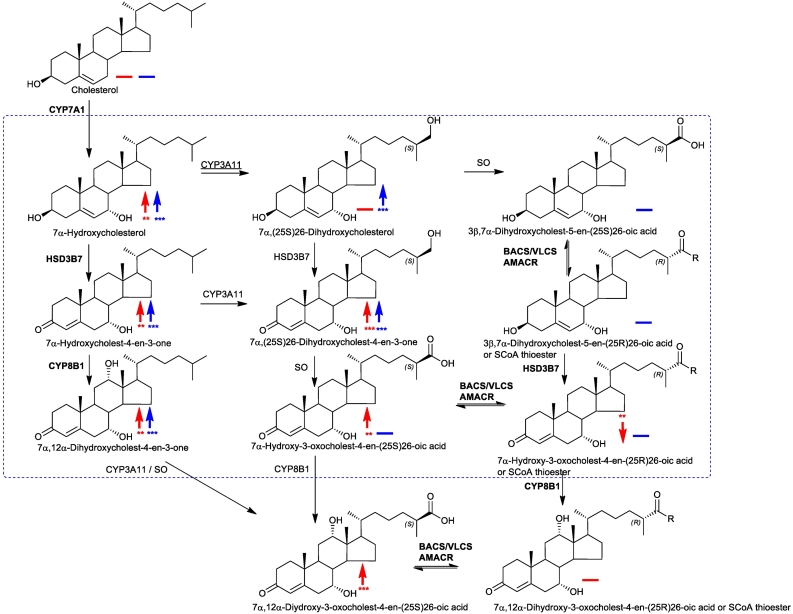
Additional routes for cholesterol metabolism in the *Cyp27a1−/−* mouse. R = H in acids or SCoA in CoA thioesters. Where known enzymes are indicated in **bold**. CYP3A11 was found in the present work to introduce a (25S)26-hydroxy group to 7α-hydroxycholesterol as indicated by underlining of the enzyme symbol. The enzyme which converts the (25S)26-primary alcohol to a carboxylic acid is indicated as a sterol oxidase (SO). Metabolites of increased or decreased abundance in the *Cyp27a1−/−* mouse are indicated by upward or downward arrow. Red arrows are used to indicate changes in plasma, blue arrows for brain. A horizontal solid line indicates detected but not changed significantly. *, *P* < 0.05; ** *P* < 0.01; *** *P* < 0.001. *P* < 0.05 is considered significant. The low levels of di- and tri-hydroxycholesterols and of dihydroxycholestenoic acids in brain makes it difficult to distinguish between these compounds and their 3-oxo equivalents using EADSA as their differentiation is based on peak area *difference* between samples treated *with* and *without* cholesterol oxidase. Hence, for these metabolites the combined values for the two structures are considered. The metabolites identified in brain are enclosed within the blue box. Abbreviations are as in Fig. 1.

Of the compounds of increased abundance in *Cyp27a1−/−* plasma or brain none activated LXR or PXR in luciferase assays performed in mouse neuronal cells, with the exception of cholest-4-en-3-one and 7α-hydroxycholest-4-en-3-one which both activated PXR (Fig. 8A, Table S2). Goodwin et al. also found that cholest-4-en-3-one and 7α-hydroxycholest-4-en-3-one both activate mouse PXR [24]. CYP3A11 in mouse and CYP3A4 in human are both PXR target genes [24,60] and Honda et al. have demonstrated that the activity of CYP3A enzyme is markedly up-regulated in the *Cyp27a1−/−* mouse [34], while both Goodwin et al. and Dussault et al. showed that hepatic expression of *Cyp3a11* is greatly increased in the *Cyp27a1−/−* mouse [24,60]. This data coupled with Honda et al.'s finding that CYP3A11 is the predominant enzyme responsible for side-chain hydroxylations of 5β-cholestane-3α,7α,12α-triol and 5β-cholestane-3α,7α,12α,25-tetrol in mouse liver microsomes [34] and our data that CYP3A11 hydroxylates 7α-hydroxycholesterol at C-26, provides *additional* routes for cholesterol metabolism in the *Cyp27a1−/−* mouse, one of which may go through (25S)26-carboxylic acids (Fig. 1, inset ii). (25S)26-CoA thioesters, generated from the corresponding acids, are substrates for the epimerase AMACR, thus the (25S)26-acids can be converted to their 25R-epimers. This is the likely explanation for the presence of both epimers in mouse plasma. Although we did not differentiate the neuroprotective molecule 3β,7α-dihydroxycholest-5-en-(25R)26-oic acid or its 25S-epimer from their 3-oxo metabolites in mouse brain from either the *Cyp27a1−/−* or the wt genotype, the current study establishes a route to the formation of the neuroprotective compound, even in the absence of CYP27A1, through up-regulated CYP3A11 as summarised in Fig. 9. It is not known whether CYP3A11, like CYP27A1, can oxidise primary alcohols to carboxylic acids, so an additional sterol oxidase may be required for the second oxidation. The *additional* pathway to 3β,7α-dihydroxycholest-5-en-(25R)26-oic acid illustrated in Fig. 9 could explain the absence of a CTX motor neuron phenotype in the *Cyp27a1−/−* mouse.

### 3.5 Effect of PXR ligands on oculomotor neurons

**Fig. 10:**
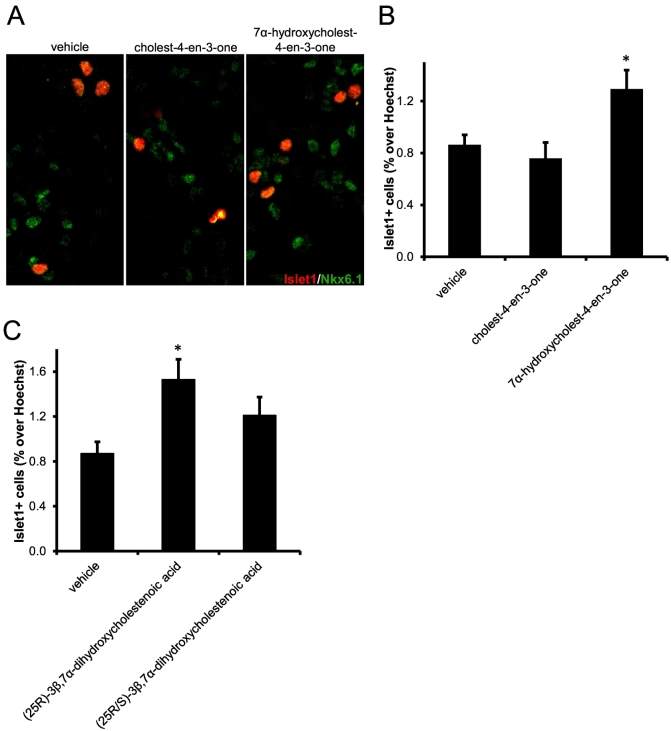
The PXR ligand 7α-hydroxycholest-4-en-3-one increases the number of Islet-1+ oculomotor neurons in mouse E11.5 midbrain primary cultures, but 3β,7α-dihydroxycholest-5-en-(25S)26-oic acid does not. (A) Representative Islet-1+ and Nkx6.1+ stained cell nuclei in cultures treated with vehicle, cholest-4-en-3-one or 7α-hydroxycholest-4-en-3-one. Quantitation of Islet-1+ neurons in primary cultures from E11.5 embryos treated with (B) vehicle, cholest-4-en-3-one or 7α-hydroxycholest-4-en-3-one, and (C) vehicle, 3β,7α-dihydroxycholest-5-en-(25R)26-oic or 3β,7α-dihydroxycholest-5-en-(25R/S)26-oic acid. * *P* < 0.05 vs vehicle treatment, Mann-Whitney test.

In our previous study of the effect of LXR ligands on oculomotor neurons we found that the LXR ligand 3β,7α-dihydroxycholest-5-en-(25R)26-oic acid increased the number of Islet-1 expressing neurons in mouse primary midbrain cultures [19]. Islet-1 is a transcription factor expressed in all postmitotic motor neurons. The increase in numbers of Islet-1+ cells was found to be a consequence of increased neuronal survival. According to the scheme presented in Fig. 9 we would predict that treatment of midbrain primary cultures with a PXR ligand would increase the synthesis of 3β,7α-dihydroxycholest-5-en-(25R)26-oic acid through up-regulated CYP3A11 and thus increase the number of Islet-1+ cells in culture. This is exactly what is observed for the more efficacious activator of PXR, 7α-hydroxycholest-4-en-3-one (Fig. 10A & B). This data substantiates the concept that neuroprotective compounds are formed in the *Cyp27a1−/−* mouse through up-regulated CYP3A11. In human, not all CTX patients suffer motor neuron signs, and perhaps the existence of an *additional* route to the neuroprotective acid through CYP3A4, the human equivalent of CYP3A11, may provide protection in these patients. Future experiments will explore the importance of these pathways in samples from CTX patients.

A further or alternative mechanism for motor neuron protection in the *Cyp27a1−/−* mouse is through 3β,7α-dihydroxycholest-5-en-(25S)26-oic acid which could have similar neuroprotective effects to its 25R-epimer. Although the two epimers can be separated by LC following derivatisation, the underivatised molecules could not be resolved chromatographically, so we investigated the effect of a commercial mixture of the two epimers (25R:25S, 3:1, mole/mol) on the number of Islet1+ cells in mouse primary brain cultures. While the 25R-epimer alone increased the number of Islet1+ cells in culture, the mixture did not (Fig. 10C), demonstrating that while 3β,7α-dihydroxycholest-5-en-(25R)26-oic acid increases the survival of oculomotor neurons, 3β,7α-dihydroxycholest-5-en-(25S)26-oic acid is less efficacious.

## 4 Conclusions

From the analysis of >50 sterols, oxysterols and sterol-acids we can conclude that a sterol hydroxylase other than CYP27A1, probably CYP3A11, is responsible for the formation of the 25S-epimers of 7α,26-dihydroxycholest-4-en-3-one, and perhaps 7α-hydroxy-3-oxcholest-4-en-26-oic and 7α,12α-dihydroxycholest-4-en-26-oic acids and their 3β-hydroxy-5-ene precursors in the *Cyp27a1−/−* mouse (Fig. 9). In this mouse the 25S-sterol-acids dominate over their 25R-epimers, while the reverse is true in the wt mice where CYP27A1 is active. In both genotypes the 25S- and 25R-sterol-acids are inter-convertible in a reaction catalysed AMACR after activation of the acids with Co-enzyme A. Thus, in the *Cyp27a1−/−* mouse an *additional* (25S)26-hydroxylase pathway may account for some of the primary bile acids formed in this mouse. It has yet to be confirmed whether CYP3A11 can convert primary alcohols to carboxylic acids in a manner similar to CYP27A1. If not, an alternative sterol oxidase must be responsible for this conversion in the *Cyp27a1−/−* mouse. One candidate is CYP24A1 which has been shown to catalyse similar reactions during vitamin D_3_ metabolism [66].

Interestingly, we also find low levels of 7α,24-dihydroxycholest-4-en-3-one in both *Cyp27a1−/−* and wt mouse brain (Figs. 5C, 6). This oxysterol has not previously been detected in brain. Its absence from plasma indicates it is formed in brain rather than being imported from the circulation.

The concurrent increase in plasma and brain of levels of many oxysterols with a 7α-hydroxycholest-4-en-3-one structure is compatible with their passage across the BBB down a concentration gradient. The current data also supports the hypothesis of others [38] that oxysterols imported to brain with a 7α-hydroxy or 7α-hydroxy-4-en-3-one structure are the precursors of cholesta-4,6-dien-3-ones in brain of *Cyp27a1−/−* mice and also humans with a similar deficiency.

We were not able to distinguish between low levels of 3β,7α-dihydroxycholest-5-en-26-oic acids and their 3-oxo-4-ene equivalents in mouse brain, but we were able to confirm their combined presence in both *Cyp27a1−/−* and wt animals. The expression in brain of the necessary enzymes to interconvert the 25R- and 25S-epimers of 3β,7α-dihydroxycholest-5-en-26-oic acids provides a biosynthetic route to the neuroprotective compound 3β,7α-dihydroxycholest-5-en-(25R)26-oic acid [18,67], providing an explanation for the lack of a motor neuron disease phenotype in the *Cyp27a1−/−* mouse.

## Conflict of interest

The EADSA technology utilised in this study is licenced to Cayman Chemical Company and Avanti Polar Lipids Inc. by Swansea Innovations, a wholly owned subsidiary of Swansea University.

The technology “Kit and method for quantitative detection of steroids”, US9851368B2, is patented by Swansea University.

## Transparency document

Transparency document Image 3

## Appendix A Supplementary data

Fig. S1 Schematic of “enzyme-assisted derivatisation for sterol analysis” EADSA. Fig. S2 Alternative routes to bile acid biosynthesis in the *Cyp27a1−/−* mouse. Abbreviations and symbols are as in Fig. 1. R = OH in acids or SCoA in CoA thioesters. Inset (i) indicates a route to 7α-hydroxydesmosterol and 7α-hydroxycholesta-4,24-dien-3-one. Inset (ii) shows the formation of cholesta-4,6-dien-3-one, 12α-hydroxycholesta-4,6-dien-3-one (R′ = OH, R″ = H) or 25-hydroxycholesta-4,6-dien-3-one (R′ = H, R″ = OH). Inset (iii) interconversion of (25S)26- and (25R)26-acids. The enzyme which converts the (25S)26-primary alcohol to a carboxylic acid is indicated as a sterol oxidase (SO). Fig. S3 Abbreviated version of the mevalonate pathway showing the Kandutsch-Russell (left), Bloch (centre) arms and the shunt pathway (right) leading to 24S,25-epoxycholesterol. R is SCoA. Abbreviations: ACAT, acetyl-CoA acetyltransferase; HMGCS, hydroxymethylglutaryl-CoA synthase; HMGCR, 3-hydroxy-3-methylglutaryl-coenzyme A reductase; SQLE, squalene epoxidase; LSS, lanosterol synthase; DHCR, dehydrocholesterol reductase; CYP cytochrome P450. Names of other enzymes and structures of further intermediates can be found at Lipidomics Gateway provided by LIPID MAPS consortium http://www.lipidmaps.org/pathways/pathway_lipids_list.php. The inset shows route to the formation of cholestanol. Fig. S4 25-Hydroxyvitamin D_3_, unsaturated and 5β-reduced oxysterols in *Cyp27a1−/−* and *Cyp27a1+/+* (wt) mouse plasma. Each chromatogram is normalised to the most intense peak at 100% RA. Magnification factors are as indicated. The concentration of the indicated analyte (by Rt) is given in the right-hand corner of each chromatogram. Chromatograms from the oxysterol fractions treated with cholesterol oxidase (combination of sterols with a *native* 3-oxo group and those *oxidised* by cholesterol oxidase to contain a 3-oxo group) are shown in (A). The chromatograms shown in (B) and (C) were generated in the absence of cholesterol oxidase enzyme. The insets show structures of generic GP derivatives. (A) 25-Hydroxyvitamin D_3_ (25-D_3_), monohydroxydehydrocholesterols and monohydroxycholesta-4,6(or24)-dien-3-ones (HCdO). (B) Monohydroxy-5β-cholestan-3-ones (H,5β-CO). (C) Dihydroxy-5β-cholestan-3-one (diH,5β-CO). Fig. S5 Cholesterol, its precursors and further unsaturated metabolites in *Cyp27a1−/−* and *Cyp27a1+/+* (wt) mouse plasma. Each chromatogram is normalised to the most intense peak at 100% RA. Magnification factors are as indicated. The concentration of the indicated analyte (by Rt) is given in the right-hand corner of each chromatogram. Chromatograms A, C and E are from the sterol fractions treated with cholesterol oxidase (combination of sterols with a *native* 3-oxo group and those *oxidised* by cholesterol oxidase to contain a 3-oxo group), chromatograms B and D are from sterol fractions not treated with cholesterol oxidase (sterols with a *native* 3-oxo group). The insets show structures of generic GP derivatives. (A) Cholesterol and cholest-4-en-3-one. (B) Cholest-4-en-3-one (CO). (C) Dehydrocholesterols (DHC) and cholestadien-3-ones. (D) Cholesta-4,6-dien-3-one (CdO). (E) Dehydrodesmosterols (DHD). Fig. S6 Concentrations of sterols in *Cyp27a1−/−* (*n* = 3) and *Cyp27a1+/+* (wt) mouse (n = 3). No hydrolysis or solvolysis steps were performed so the values reported are for “free” non-esterified molecules. (A) sterols in plasma, (B) sterols in brain. To maintain a single y-axis magnification factors have been applied as indicated. Fig. S7 Routes to formation of, or import of, monohydroxycholesterols and dihydroxycholest-4-en-3-ones in brain. Abbreviations and symbols are as in Fig. 1. Inset (i) indicates the formation of 12α-hydroxycholesterol, (ii) a route to 7α,(25S)26-dihydroxycholest-4-en-3-one, (iii) a route to 7α,12α-dihydroxycholest-4-en-3-one, and (iv) the formation of 7β-hydroxycholesterol. Where known, enzymes are indicated in **bold**. CYP3A11 was found in the present work to introduce a (25S)26-hydroxy or 25-hydroxy group to 7α-hydroxycholesterol as indicated by underlining of the enzyme symbol. Fig. S8 Cholesterol, its precursors and further unsaturated metabolites in *Cyp27a1−/−* and *Cyp27a1+/+* (wt) mouse brain. Each chromatogram is normalised to the most intense peak at 100% RA. Magnification factors are as indicated. The concentration of the indicated analyte (by Rt) is given in the right-hand corner of each chromatogram. Chromatograms (A) and (C) are from the sterol fractions treated with cholesterol oxidase (combination of sterols with a *native* 3-oxo group and those *oxidised* by cholesterol oxidase to contain a 3-oxo group), chromatograms (B) and (D) are from sterol fractions not treated with cholesterol oxidase (sterols with a *native* 3-oxo group). The insets show structures of generic GP derivatives. (A) Cholesterol and cholest-4-en-3-one. (B) Cholest-4-en-3-one (CO). (C) Dehydrocholesterols (DHC) and cholestadien-3-ones. (D) Cholesta-4,6-dien-3-one (CdO). Image 1

Supplementary tables Image 2
