# Supplementary material for: Additional pathways of sterol metabolism: Evidence from analysis of Cyp27a1−/− mouse brain and plasma
Source: Biochim Biophys Acta Mol Cell Biol Lipids. 2019 Feb;1864(2):191–211. doi: 10.1016/j.bbalip.2018.11.006 (PMC6327153; doi:10.1016/j.bbalip.2018.11.006)
Supplement: Supplementary file 2 — Fig. S1 Schematic of “enzyme-assisted derivatisation for sterol analysis” EADSA. Fig. S2 Alternative routes to bile acid biosynthesis in the Cyp27a1−/− mouse. Abbreviations and symbols are as in Fig. 1. R = OH in acids or SCoA in CoA thioesters. Inset (i) indicates a route to 7α-hydroxydesmosterol and 7α-hydroxycholesta-4,24-dien-3-one. Inset (ii) shows the formation of cholesta-4,6-dien-3-one, 12α-hydroxycholesta-4,6-dien-3-one (R′ = OH, R″ = H) or 25-hydroxycholesta-4,6-dien-3-one (R′ = H, R″ = OH). Inset (iii) interconversion of (25S)26- and (25R)26-acids. The enzyme which converts the (25S)26-primary alcohol to a carboxylic acid is indicated as a sterol oxidase (SO). Fig. S3 Abbreviated version of the mevalonate pathway showing the Kandutsch-Russell (left), Bloch (centre) arms and the shunt pathway (right) leading to 24S,25-epoxycholesterol. R is SCoA. Abbreviations: ACAT, acetyl-CoA acetyltransferase; HMGCS, hydroxymethylglutaryl-CoA synthase; HMGCR, 3-hydroxy-3-methylglutaryl-coenzyme A reductase; SQLE, squalene epoxidase; LSS, lanosterol synthase; DHCR, dehydrocholesterol reductase; CYP cytochrome P450. Names of other enzymes and structures of further intermediates can be found at Lipidomics Gateway provided by LIPID MAPS consortium http://www.lipidmaps.org/pathways/pathway_lipids_list.php. The inset shows route to the formation of cholestanol. Fig. S4 25-Hydroxyvitamin D3, unsaturated and 5β-reduced oxysterols in Cyp27a1−/− and Cyp27a1+/+ (wt) mouse plasma. Each chromatogram is normalised to the most intense peak at 100% RA. Magnification factors are as indicated. The concentration of the indicated analyte (by Rt) is given in the right-hand corner of each chromatogram. Chromatograms from the oxysterol fractions treated with cholesterol oxidase (combination of sterols with a native 3-oxo group and those oxidised by cholesterol oxidase to contain a 3-oxo group) are shown in (A). The chromatograms shown in (B) and (C) were generated in the absence of cholestero [file mmc1.pdf]

S1

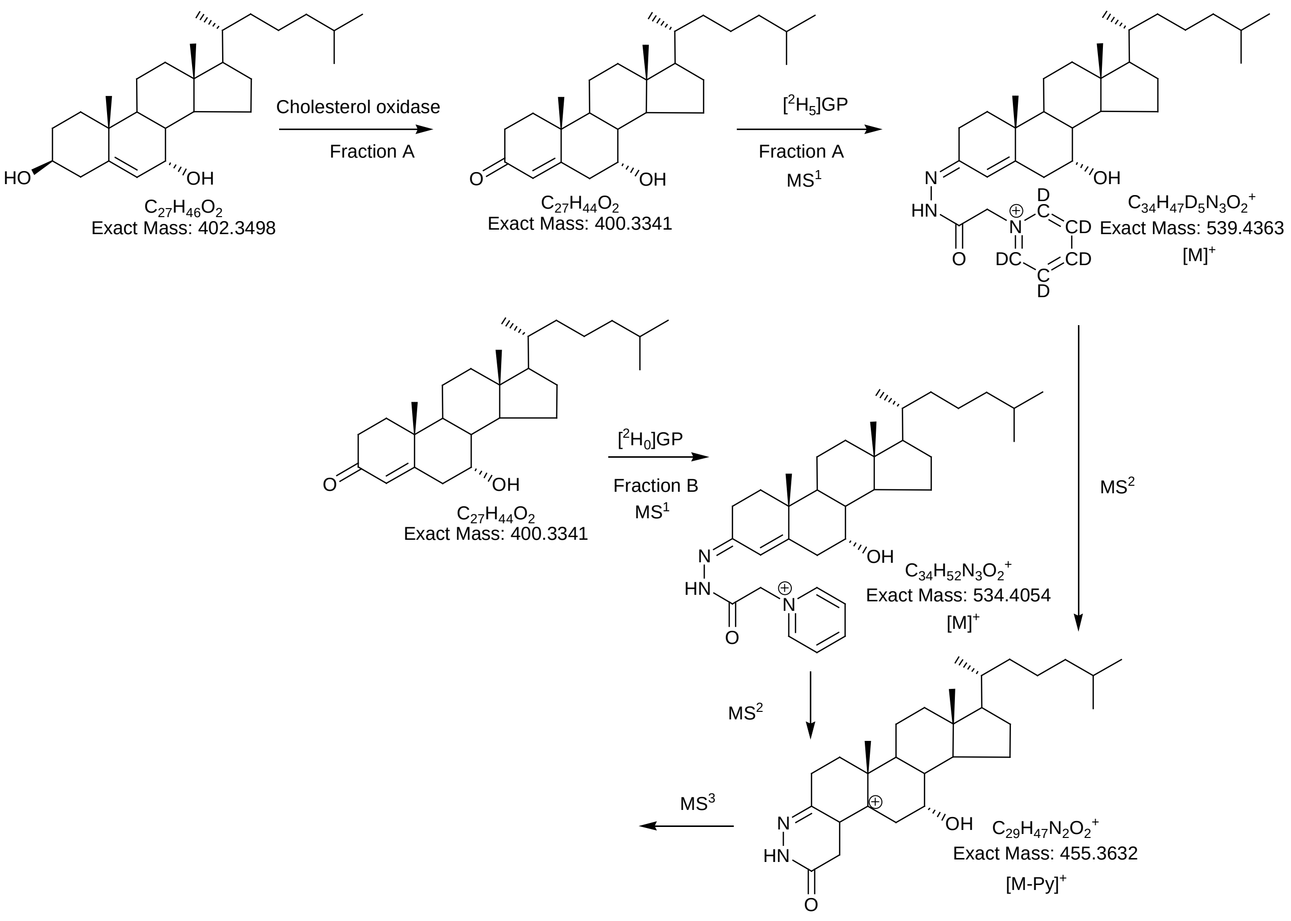

HO
OH
Cholesterol oxidase
Fraction A
O
OH
[2H5]GP
Fraction A
MS1
N
HN
O
D
C
N
CD
DC
CD
D
OH
C27H46O2
Exact Mass: 402.3498
C27H44O2
Exact Mass: 400.3341
C34H47D5N3O2+
Exact Mass: 539.4363
[M]+
O
OH
C27H44O2
Exact Mass: 400.3341
[2H0]GP
Fraction B
MS1
N
HN
O
N
OH
C34H52N3O2+
Exact Mass: 534.4054
[M]+
MS2
MS2
MS3
N
HN
O
OH
C29H47N2O2+
Exact Mass: 455.3632
[M-Py]+

S2

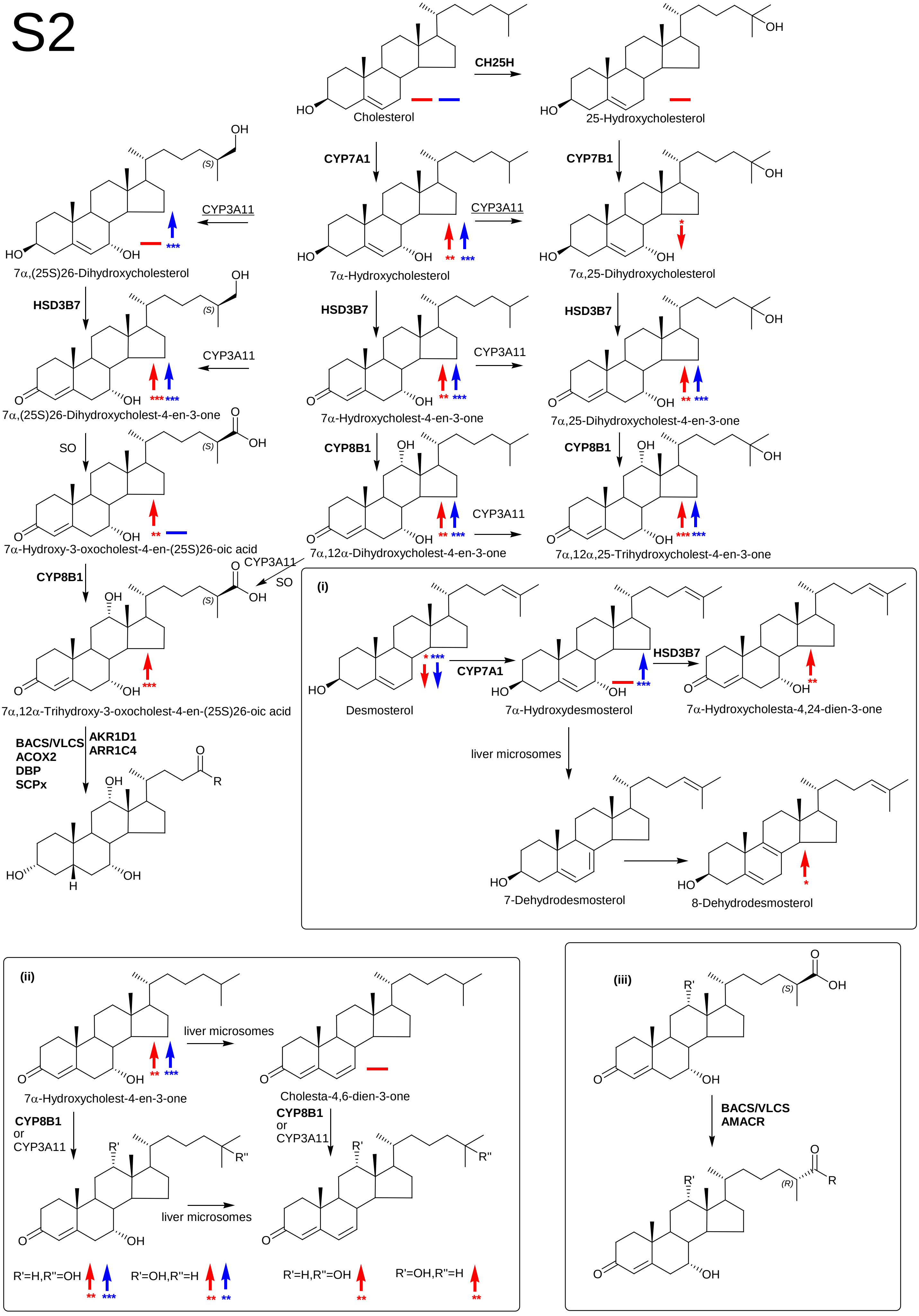

Cholesterol
CH25H
25-Hydroxycholesterol
CYP7A1
CYP7B1
CYP3A11
7α,(25S)26-Dihydroxycholesterol
7α-Hydroxycholesterol
7α,25-Dihydroxycholesterol
HSD3B7
7α,(25S)26-Dihydroxycholest-4-en-3-one
7α-Hydroxycholest-4-en-3-one
7α,25-Dihydroxycholest-4-en-3-one
SO
CYP8B1
7α-Hydroxy-3-oxocholest-4-en-(25S)26-oic acid
7α,12α-Dihydroxycholest-4-en-3-one
7α,12α,25-Trihydroxycholest-4-en-3-one
7α,12α-Trihydroxy-3-oxocholest-4-en-(25S)26-oic acid
BACS/VLCS
ACOX2
DBP
SCPx
AKR1D1
ARR1C4
(i)
Desmosterol
7α-Hydroxydesmosterol
7α-Hydroxycholesta-4,24-dien-3-one
liver microsomes
7-Dehydrodesmosterol
8-Dehydrodesmosterol
(ii)
7α-Hydroxycholest-4-en-3-one
Cholesta-4,6-dien-3-one
CYP8B1
or
CYP3A11
R'=H,R"=OH
R'=OH,R"=H
(iii)
BACS/VLCS
AMACR

S3

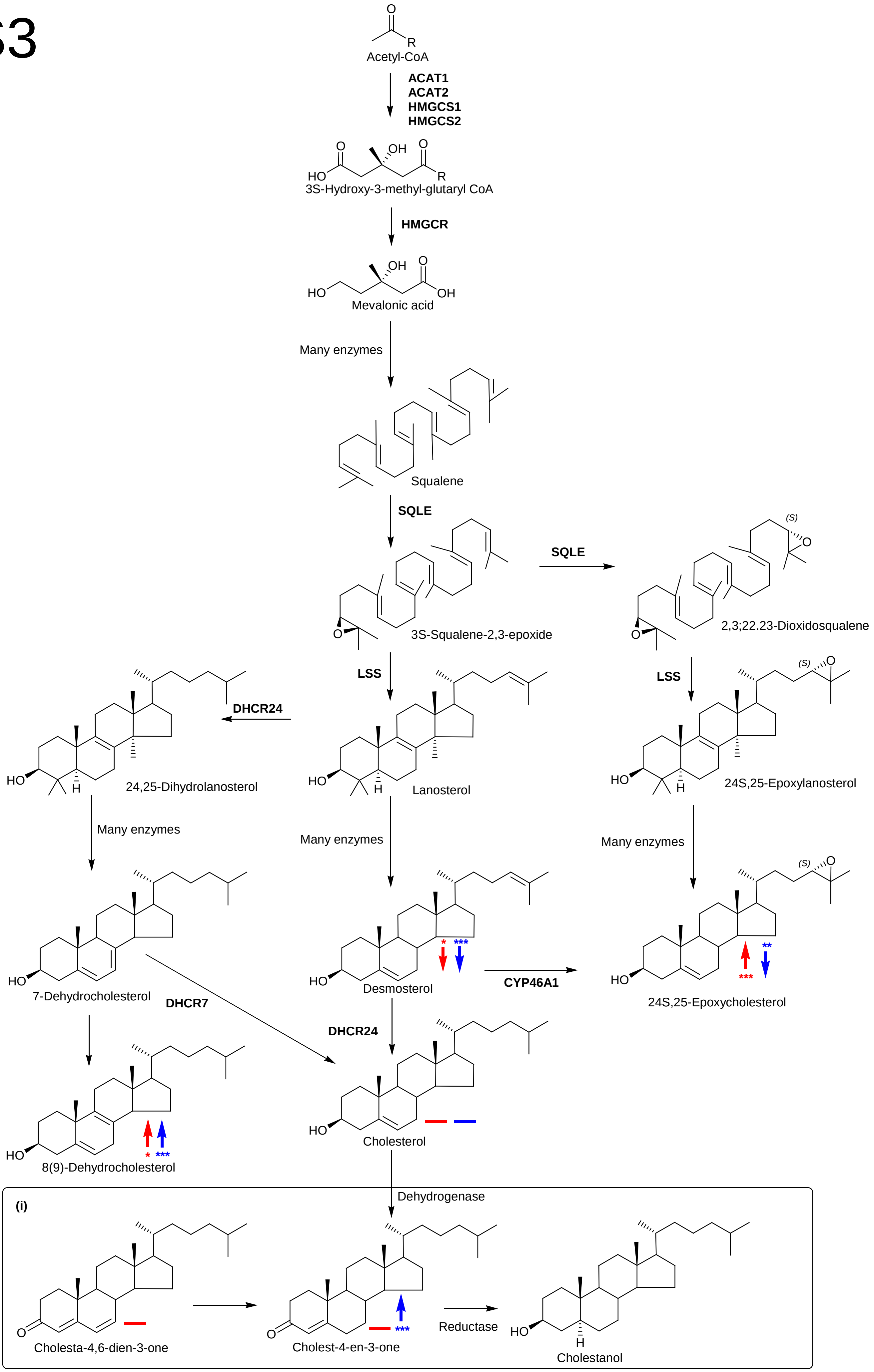

Acetyl-CoA
ACAT1
ACAT2
HMGCS1
HMGCS2
3S-Hydroxy-3-methyl-glutaryl CoA
HMGCR
Mevalonic acid
Many enzymes
Squalene
SQLE
3S-Squalene-2,3-epoxide
SQLE
2,3;22.23-Dioxidosqualene
LSS
Lanosterol
DHCR24
24,25-Dihydrolanosterol
LSS
24S,25-Epoxylanosterol
Many enzymes
7-Dehydrocholesterol
Many enzymes
Desmosterol
Many enzymes
24S,25-Epoxycholesterol
CYP46A1
DHCR7
DHCR24
8(9)-Dehydrocholesterol
Cholesterol
Dehydrogenase
(i)
Cholesta-4,6-dien-3-one
Cholest-4-en-3-one
Reductase
Cholestanol

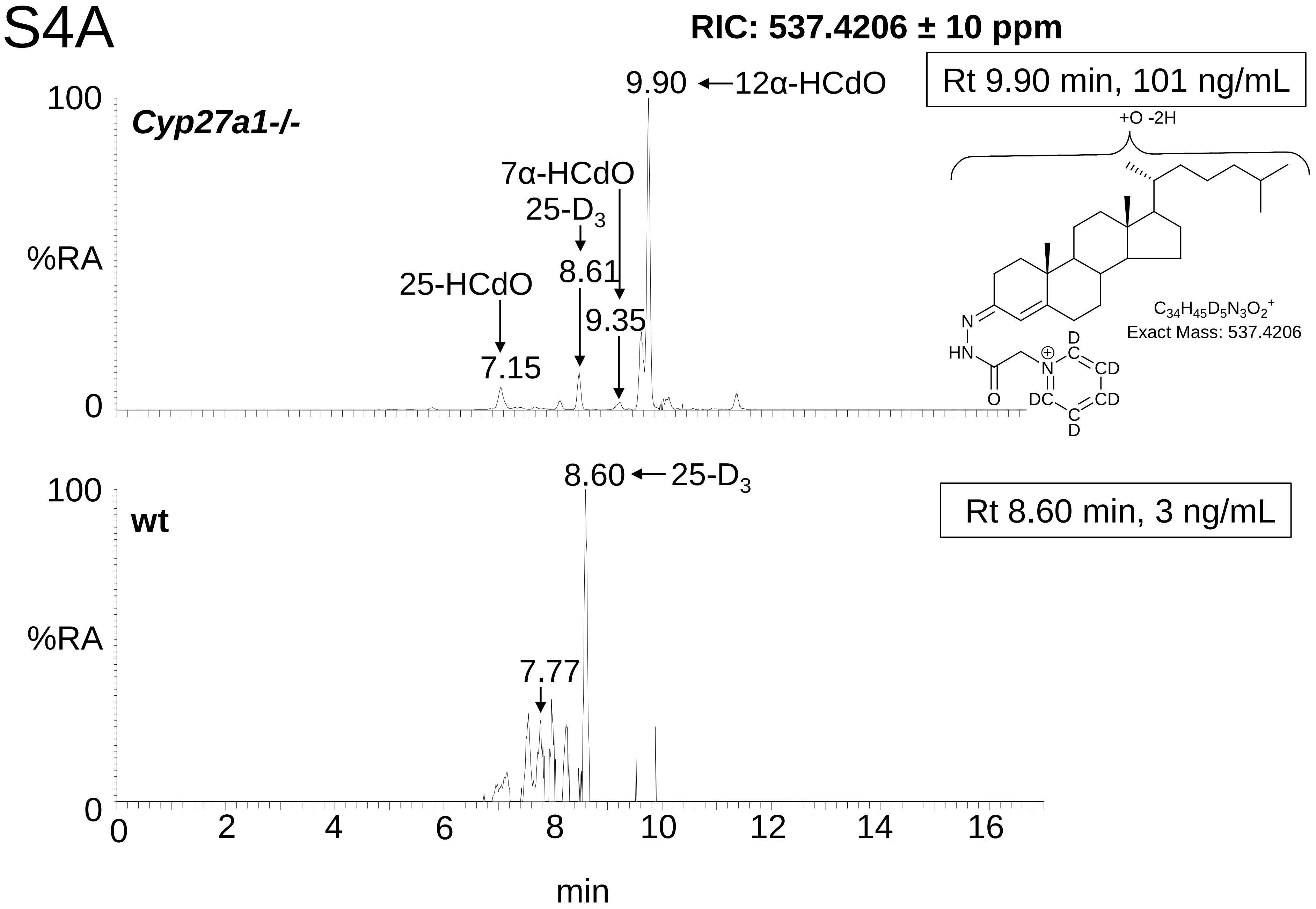
S4A
RIC: 537.4206 ± 10 ppm
Rt 9.90 min, 101 ng/mL
9.90
12α-HCdO
Cyp27a1-/-
7α-HCdO
25-D3
8.61
25-HCdO
9.35
7.15
100
%RA
0
+O -2H
C34H45D5N3O2+
Exact Mass: 537.4206
8.60
25-D3
Rt 8.60 min, 3 ng/mL
wt
7.77
0
2
4
6
8
10
12
14
16
min

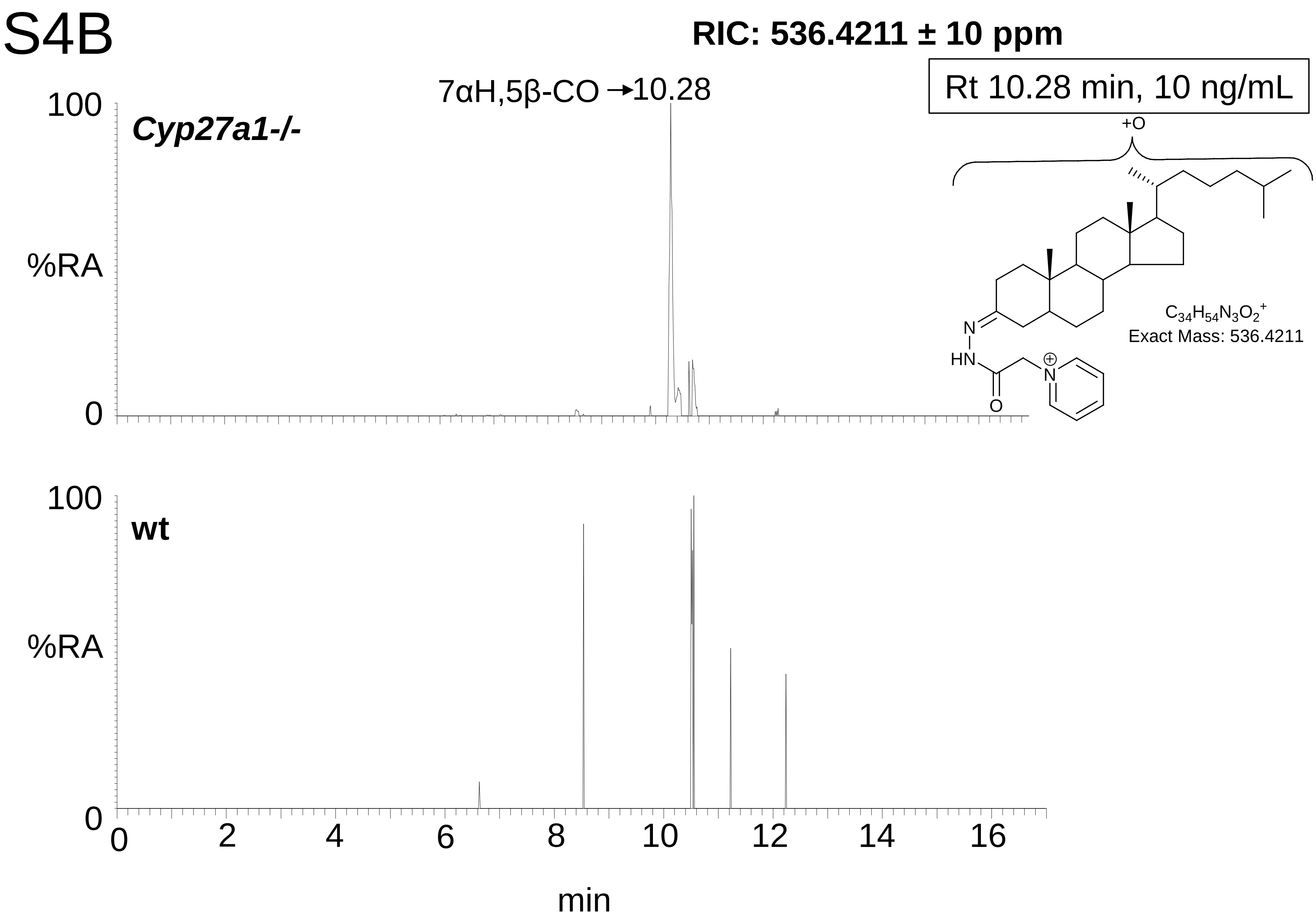
S4B
RIC: 536.4211 ± 10 ppm
Rt 10.28 min, 10 ng/mL
7αH,5β-CO →10.28
Cyp27a1-/-
wt
100
%RA
0
0
2
4
6
8
10
12
14
16
min
+O
N
HN
N
O
$C_{34}H_{54}N_3O_2^+$
Exact Mass: 536.4211

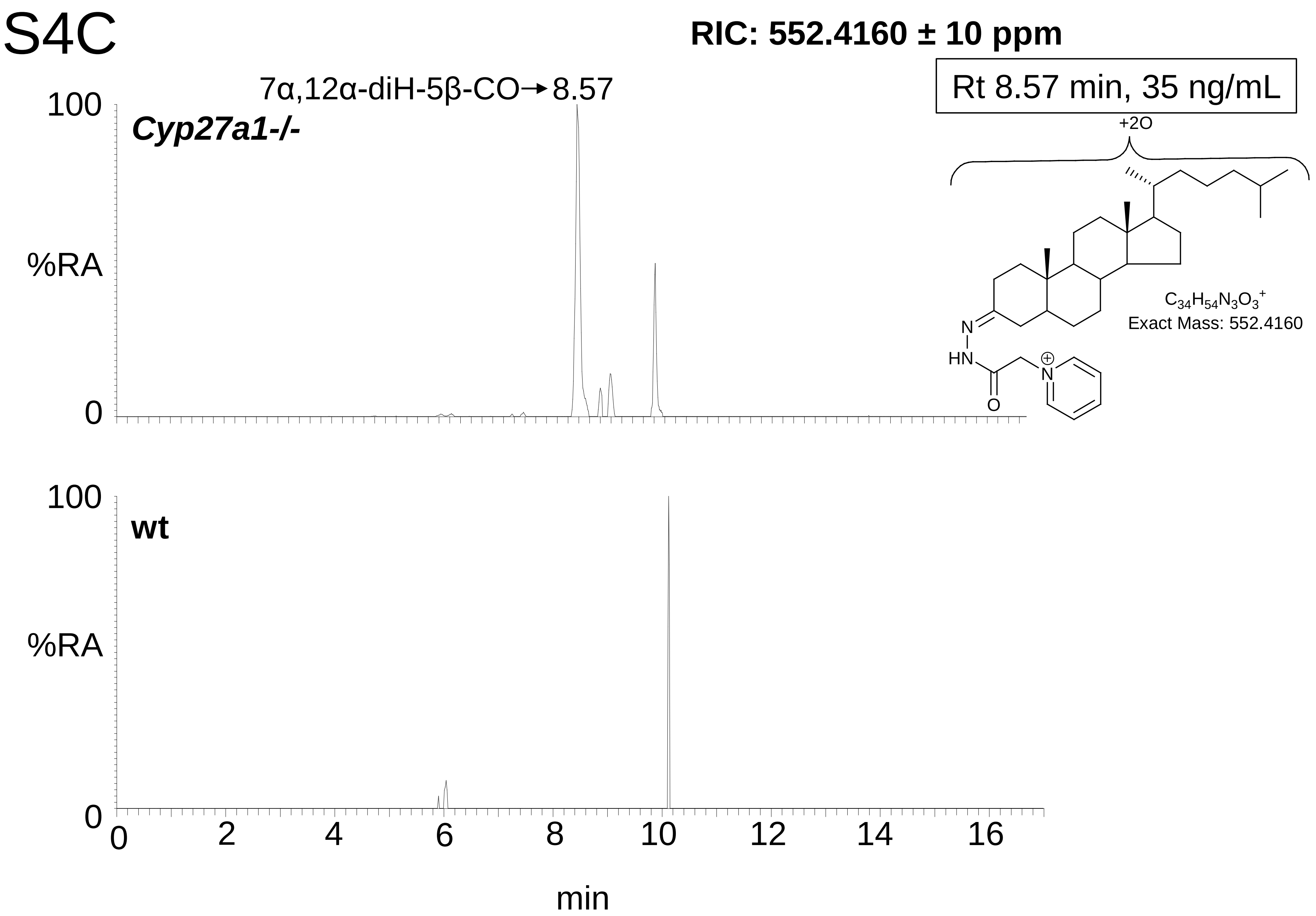
S4C
RIC: 552.4160 ± 10 ppm
Rt 8.57 min, 35 ng/mL
7α,12α-diH-5β-CO→8.57
100
Cyp27a1-/-
%RA
0
+2O
$C_{34}H_{54}N_3O_3^+$
Exact Mass: 552.4160
N
HN
N
O
100
wt
%RA
0
0
2
4
6
8
10
12
14
16
min

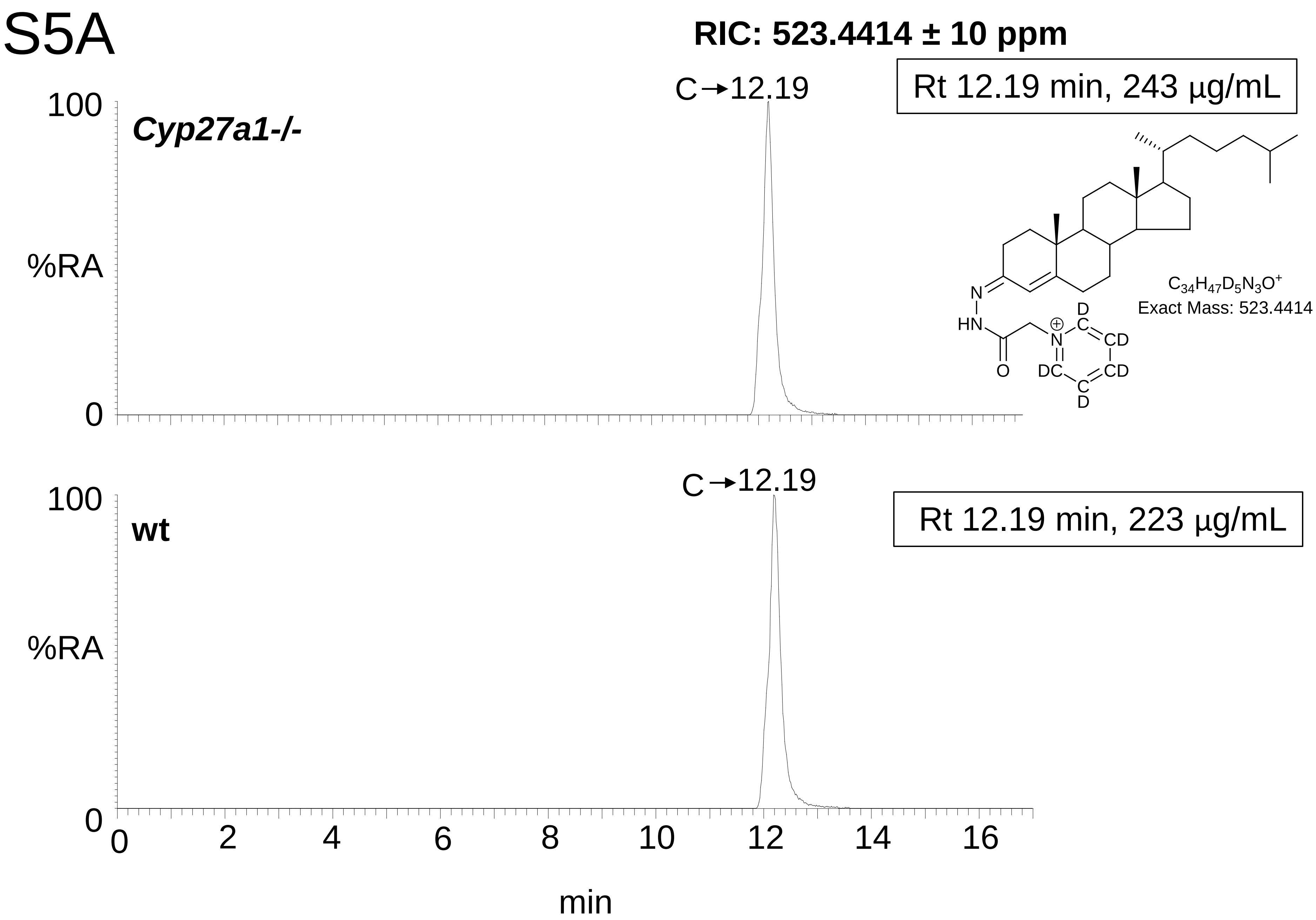
S5A
RIC: 523.4414 ± 10 ppm
C→12.19
Rt 12.19 min, 243 µg/mL
Cyp27a1-/-
100
%RA
0
C34H47D5N3O+
Exact Mass: 523.4414
C→12.19
Rt 12.19 min, 223 µg/mL
wt
100
%RA
0
0
2
4
6
8
10
12
14
16
min

S5B

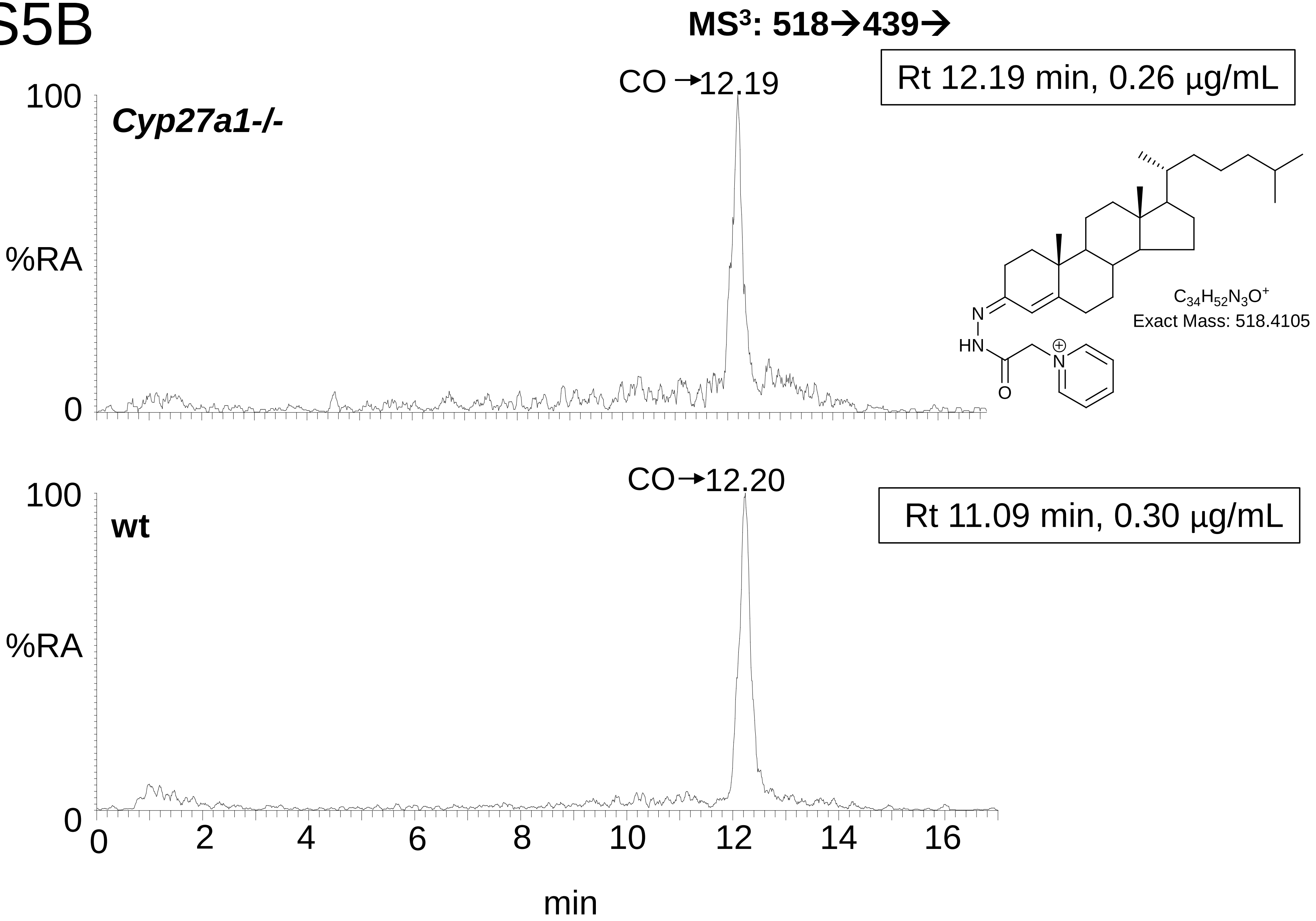

MS³: 518→439→
CO→12.19
Rt 12.19 min, 0.26 µg/mL
100
Cyp27a1-/-
%RA
0
N
HN
O
N
C34H52N3O+
Exact Mass: 518.4105
CO→12.20
Rt 11.09 min, 0.30 µg/mL
100
wt
%RA
0
0
2
4
6
8
10
12
14
16
min

S5C

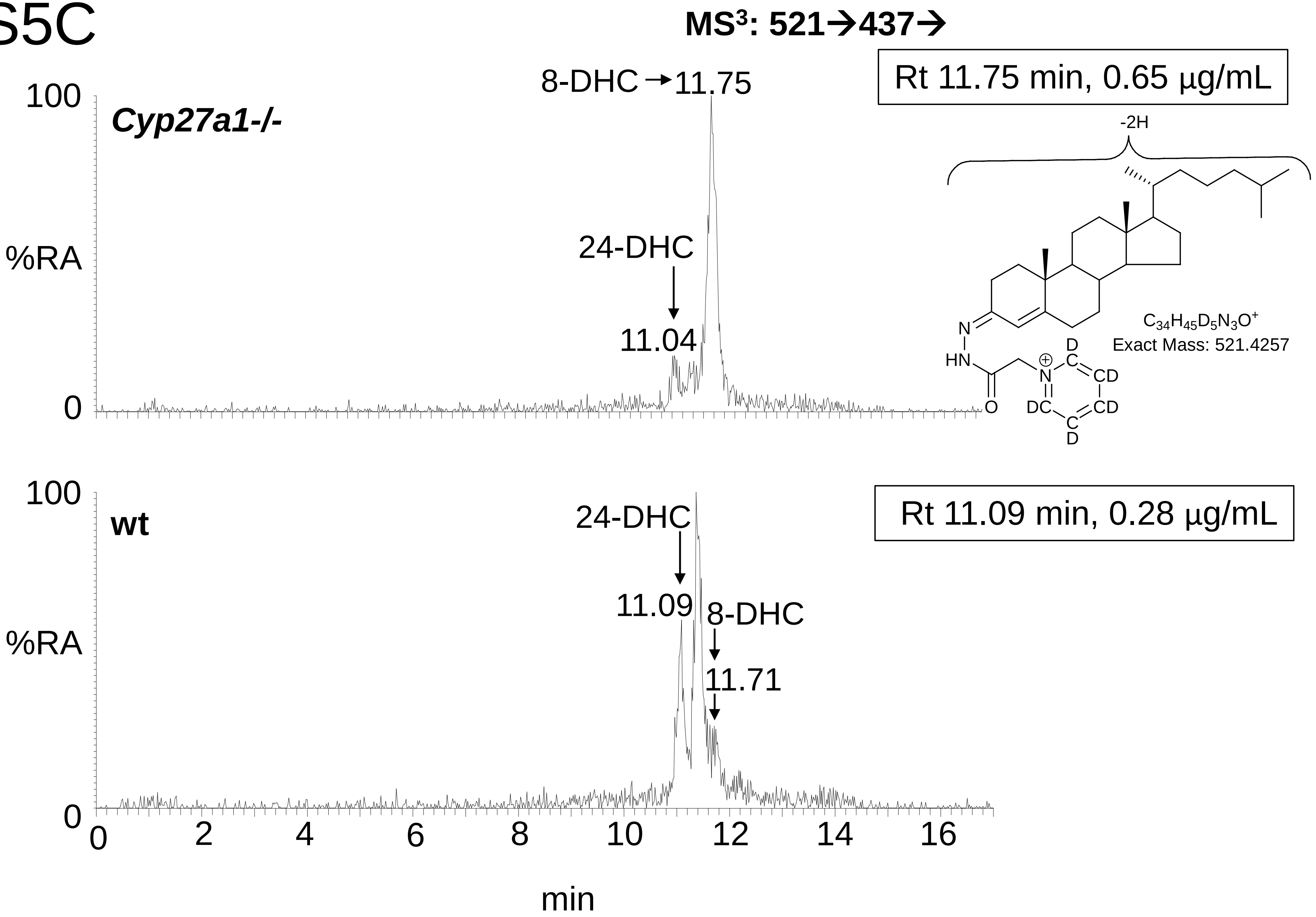
MS3: 521→437→
Rt 11.75 min, 0.65 µg/mL
8-DHC →11.75
Cyp27a1-/-
24-DHC
11.04
100
%RA
0
-2H
C34H45D5N3O+
Exact Mass: 521.4257
N
HN
O
N
D
C
CD
CD
DC
C
D
wt
Rt 11.09 min, 0.28 µg/mL
24-DHC
11.09
8-DHC
11.71
100
%RA
0
0
2
4
6
8
10
12
14
16
min

S5D

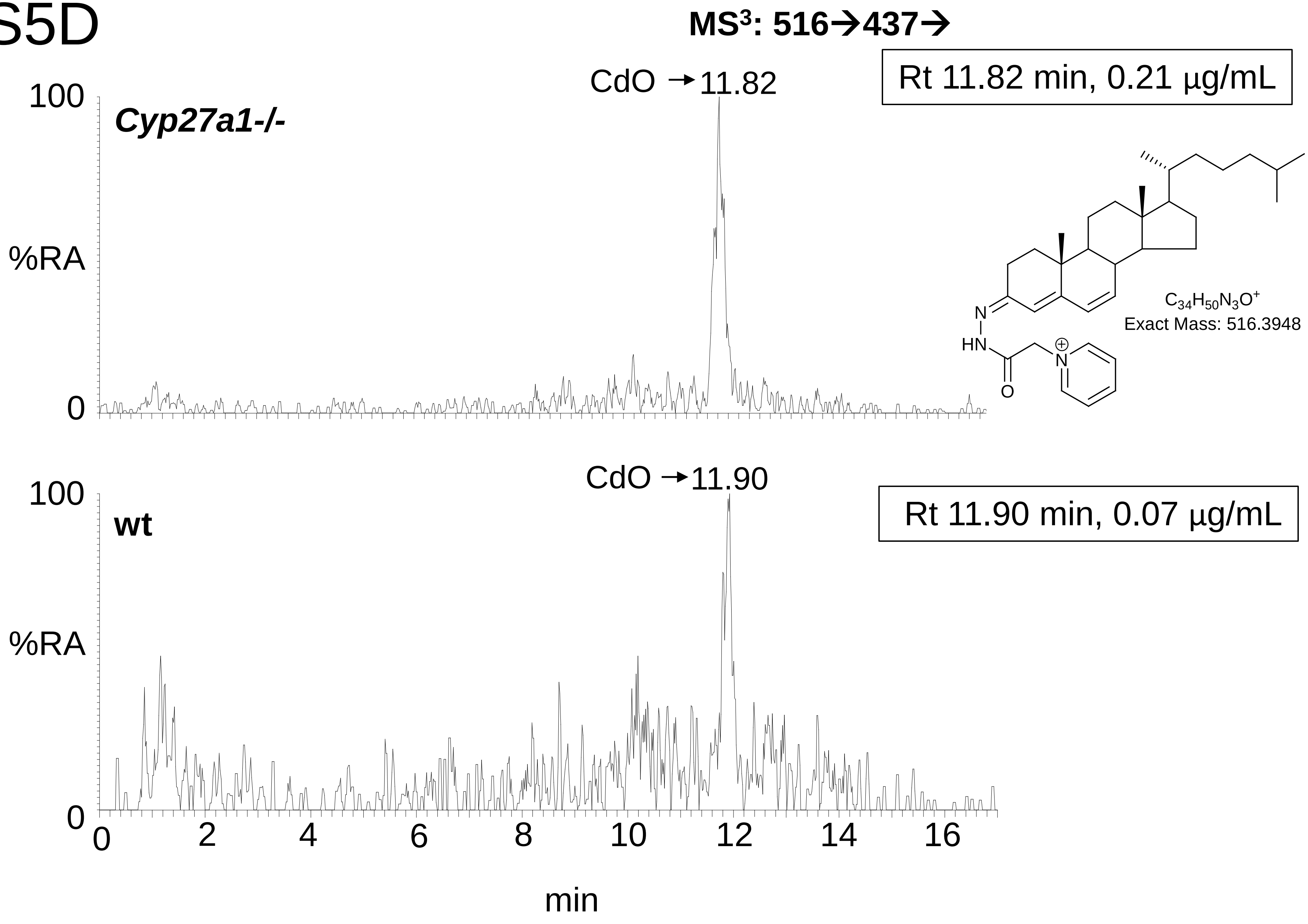

MS³: 516→437→
CdO →11.82
Rt 11.82 min, 0.21 µg/mL
100
Cyp27a1-/-
%RA
0
N
HN
O
N
C34H50N3O+
Exact Mass: 516.3948
CdO →11.90
Rt 11.90 min, 0.07 µg/mL
100
wt
%RA
0
0
2
4
6
8
10
12
14
16
min

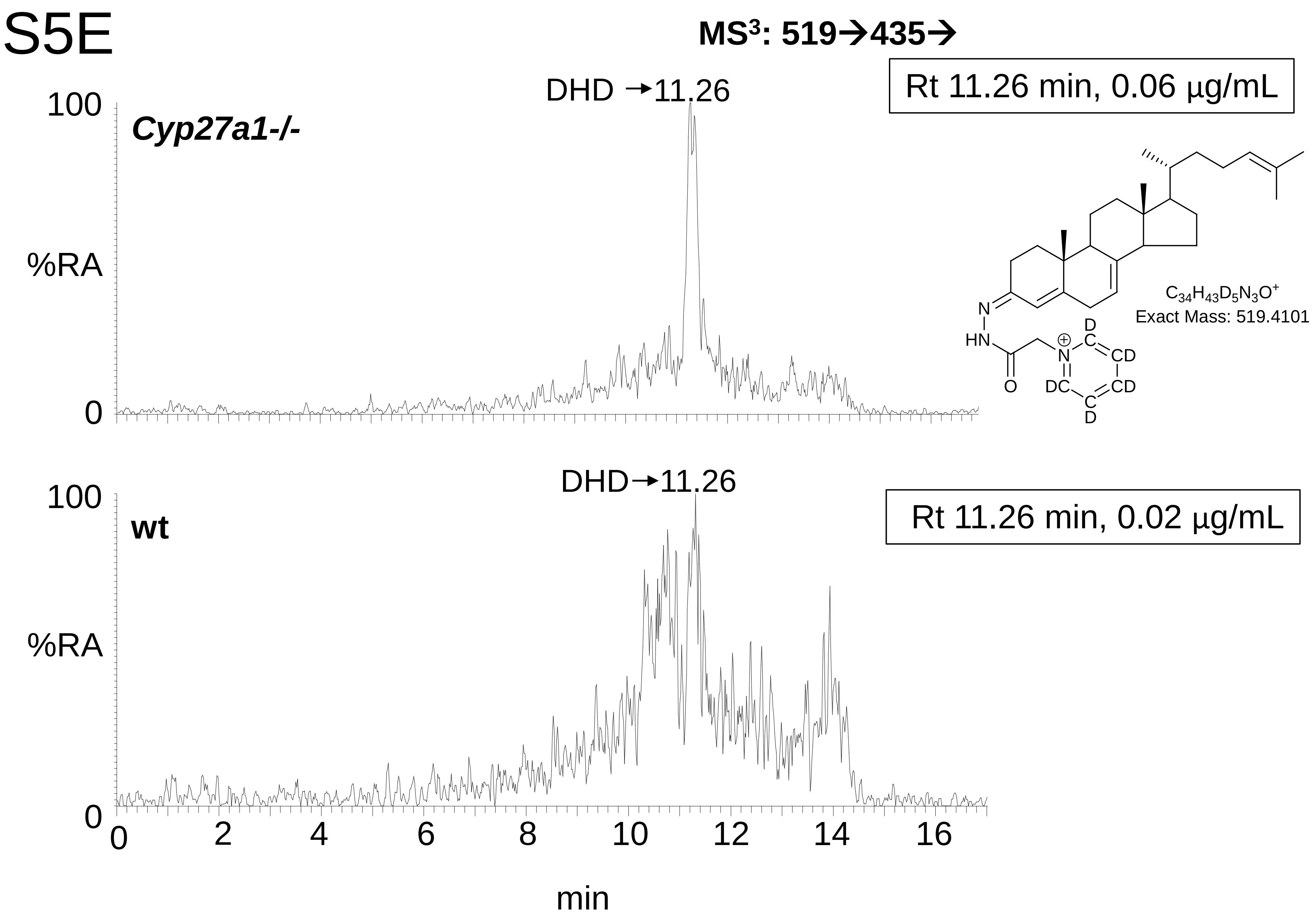
S5E
MS³: 519→435→
DHD →11.26
Rt 11.26 min, 0.06 µg/mL
100
Cyp27a1-/-
%RA
0
C34H43D5N3O+
Exact Mass: 519.4101
DHD→11.26
Rt 11.26 min, 0.02 µg/mL
100
wt
%RA
0
0
2
4
6
8
10
12
14
16
min

S6A

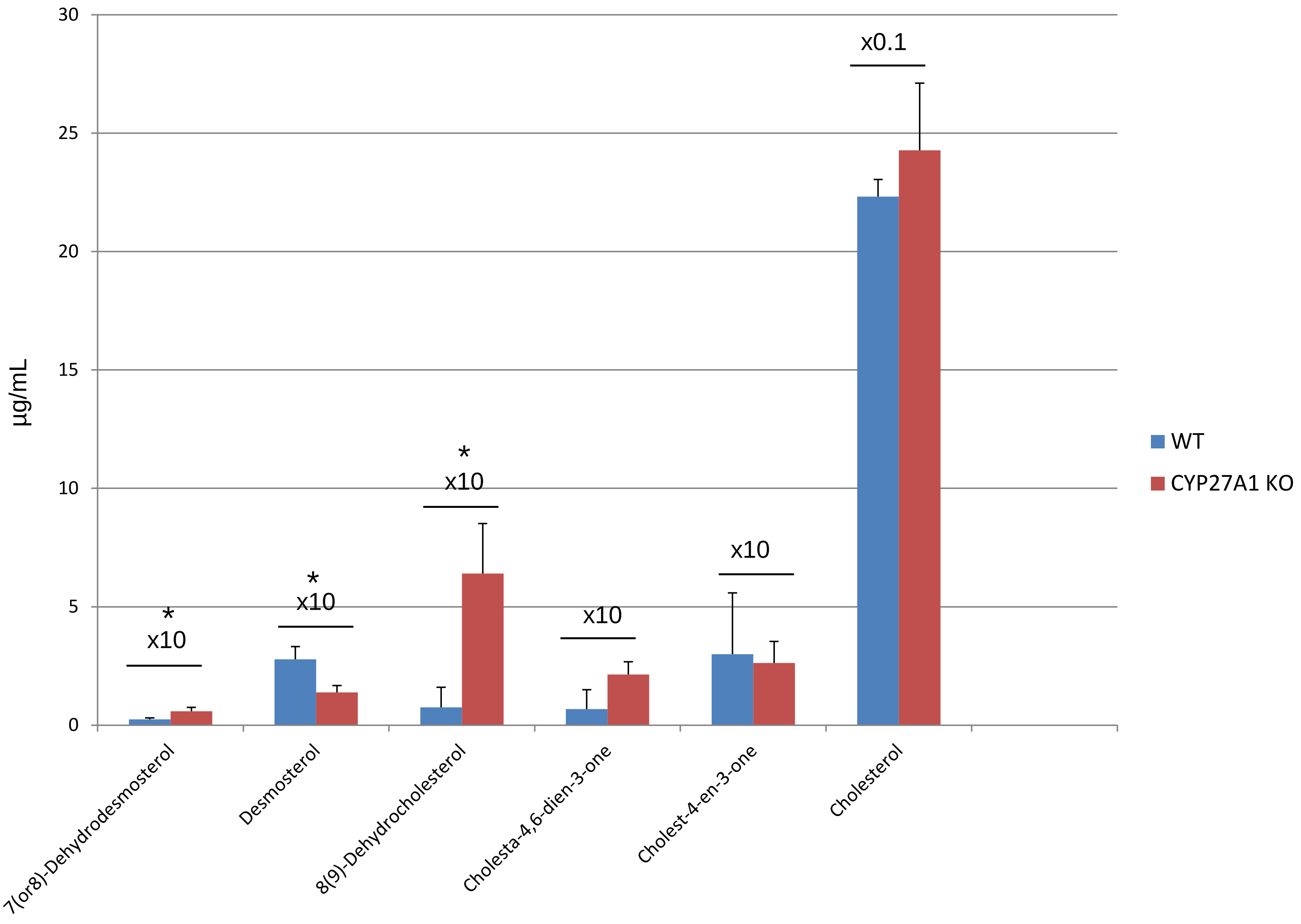

30
25
20
15
10
5
0
µg/mL
x0.1
*
x10
*
x10
*
x10
x10
x10
WT
CYP27A1 KO
7(or8)-Dehydrodesmosterol
Desmosterol
8(9)-Dehydrocholesterol
Cholesta-4,6-dien-3-one
Cholest-4-en-3-one
Cholesterol

S6B

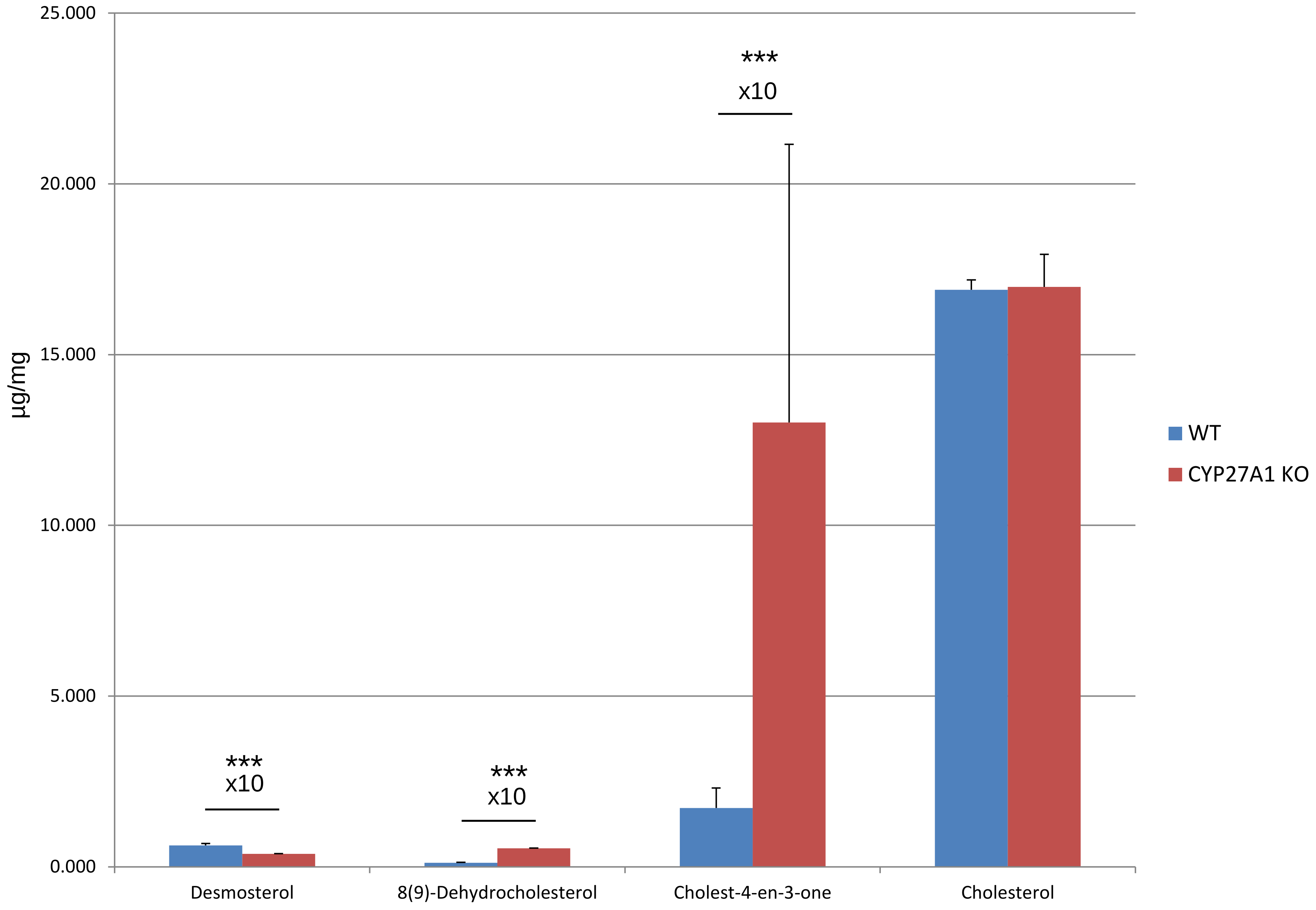

25.000
20.000
15.000
10.000
5.000
0.000
µg/mg
***
x10
***
x10
***
x10
Desmosterol
8(9)-Dehydrocholesterol
Cholest-4-en-3-one
Cholesterol
WT
CYP27A1 KO

S7

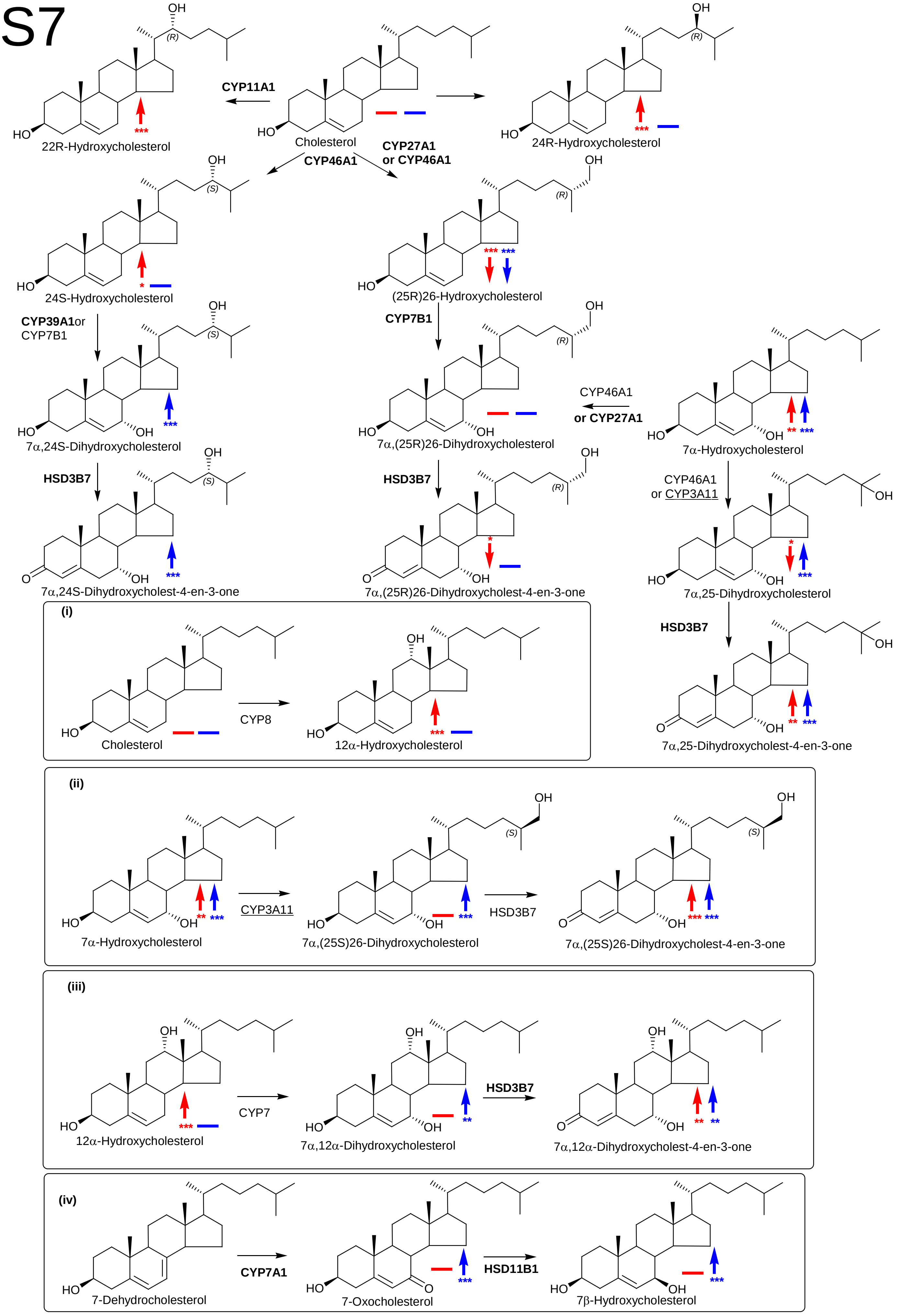

22R-Hydroxycholesterol
CYP11A1
Cholesterol
24R-Hydroxycholesterol
CYP46A1
CYP27A1
or CYP46A1
24S-Hydroxycholesterol
(25R)26-Hydroxycholesterol
CYP39A1or
CYP7B1
CYP7B1
7α,24S-Dihydroxycholesterol
7α,(25R)26-Dihydroxycholesterol
CYP46A1
or CYP27A1
7α-Hydroxycholesterol
HSD3B7
HSD3B7
CYP46A1
or CYP3A11
7α,24S-Dihydroxycholest-4-en-3-one
7α,(25R)26-Dihydroxycholest-4-en-3-one
7α,25-Dihydroxycholesterol
HSD3B7
(i)
Cholesterol
CYP8
12α-Hydroxycholesterol
7α,25-Dihydroxycholest-4-en-3-one
(ii)
7α-Hydroxycholesterol
CYP3A11
7α,(25S)26-Dihydroxycholesterol
HSD3B7
7α,(25S)26-Dihydroxycholest-4-en-3-one
(iii)
12α-Hydroxycholesterol
CYP7
7α,12α-Dihydroxycholesterol
HSD3B7
7α,12α-Dihydroxycholest-4-en-3-one
(iv)
7-Dehydrocholesterol
CYP7A1
7-Oxocholesterol
HSD11B1
7β-Hydroxycholesterol

S8A

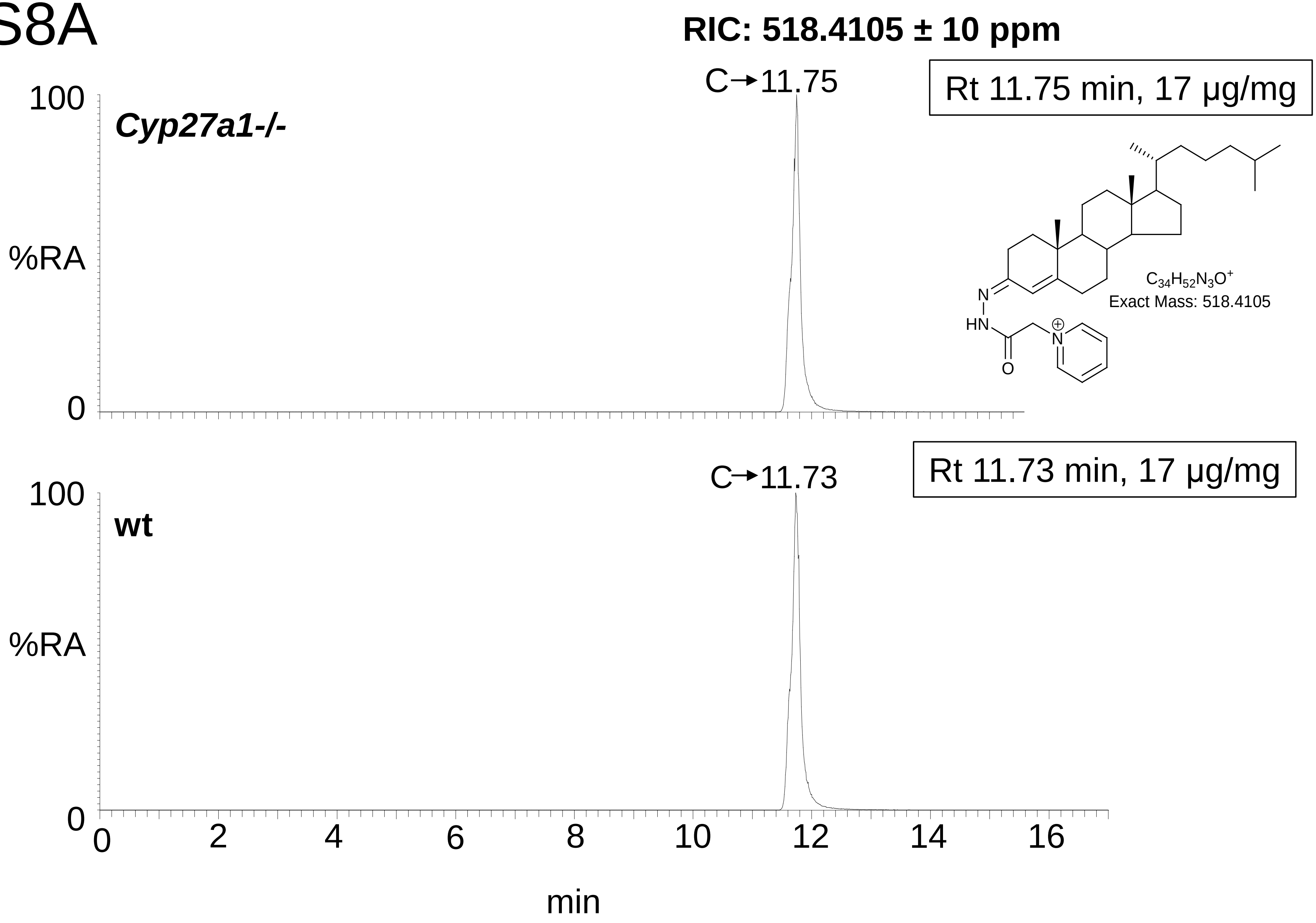
RIC: 518.4105 ± 10 ppm
C→11.75
Rt 11.75 min, 17 µg/mg
100
Cyp27a1-/-
%RA
0
$C_{34}H_{52}N_3O^+$
Exact Mass: 518.4105
N
HN
N
O
C→11.73
Rt 11.73 min, 17 µg/mg
100
wt
%RA
0
0
2
4
6
8
10
12
14
16
min

S8B

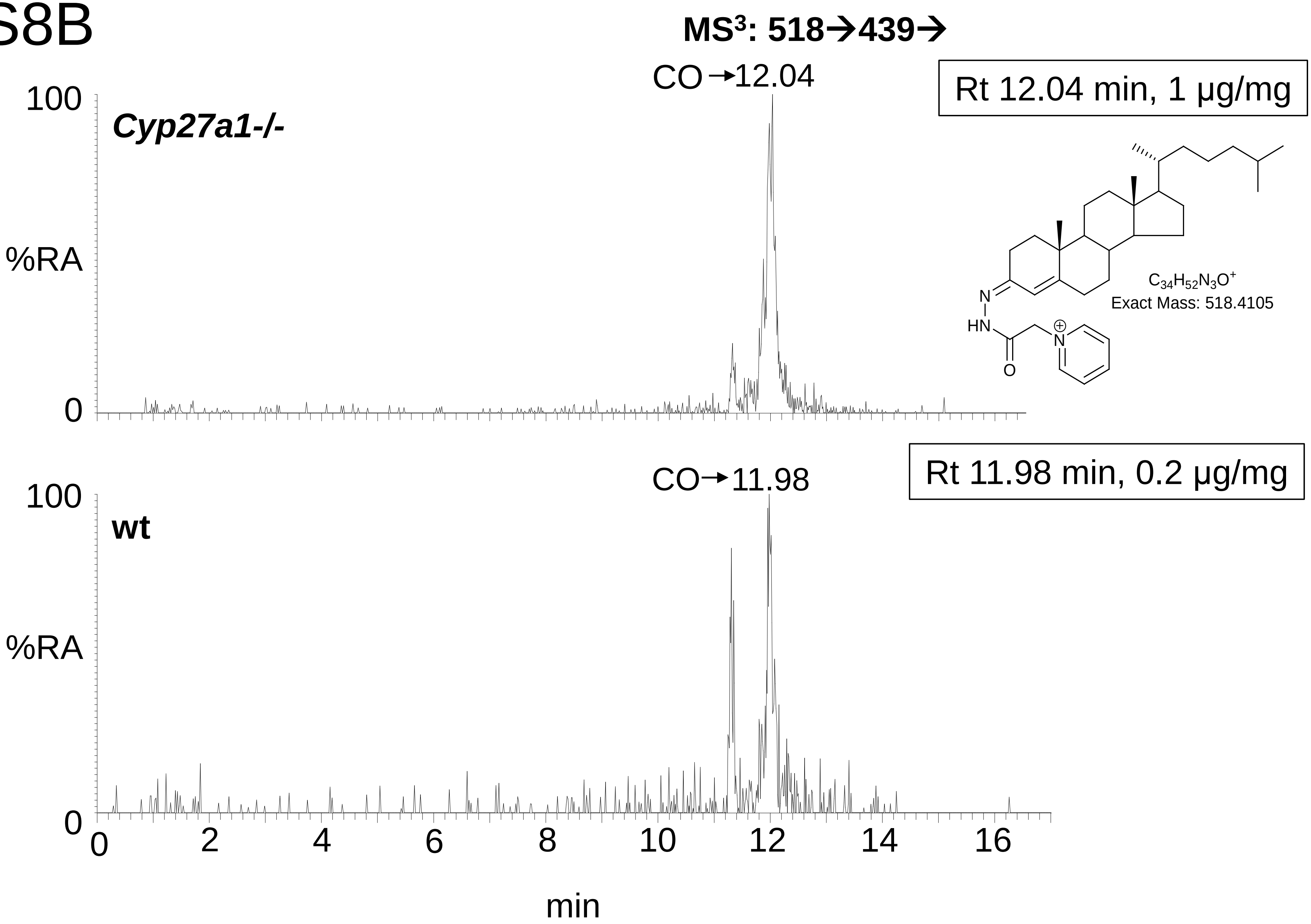

MS3: 518→439→
CO→12.04
Rt 12.04 min, 1 µg/mg
100
Cyp27a1-/-
%RA
0
C34H52N3O+
Exact Mass: 518.4105
N
HN
N
O
CO→11.98
Rt 11.98 min, 0.2 µg/mg
100
wt
%RA
0
0
2
4
6
8
10
12
14
16
min

S8C

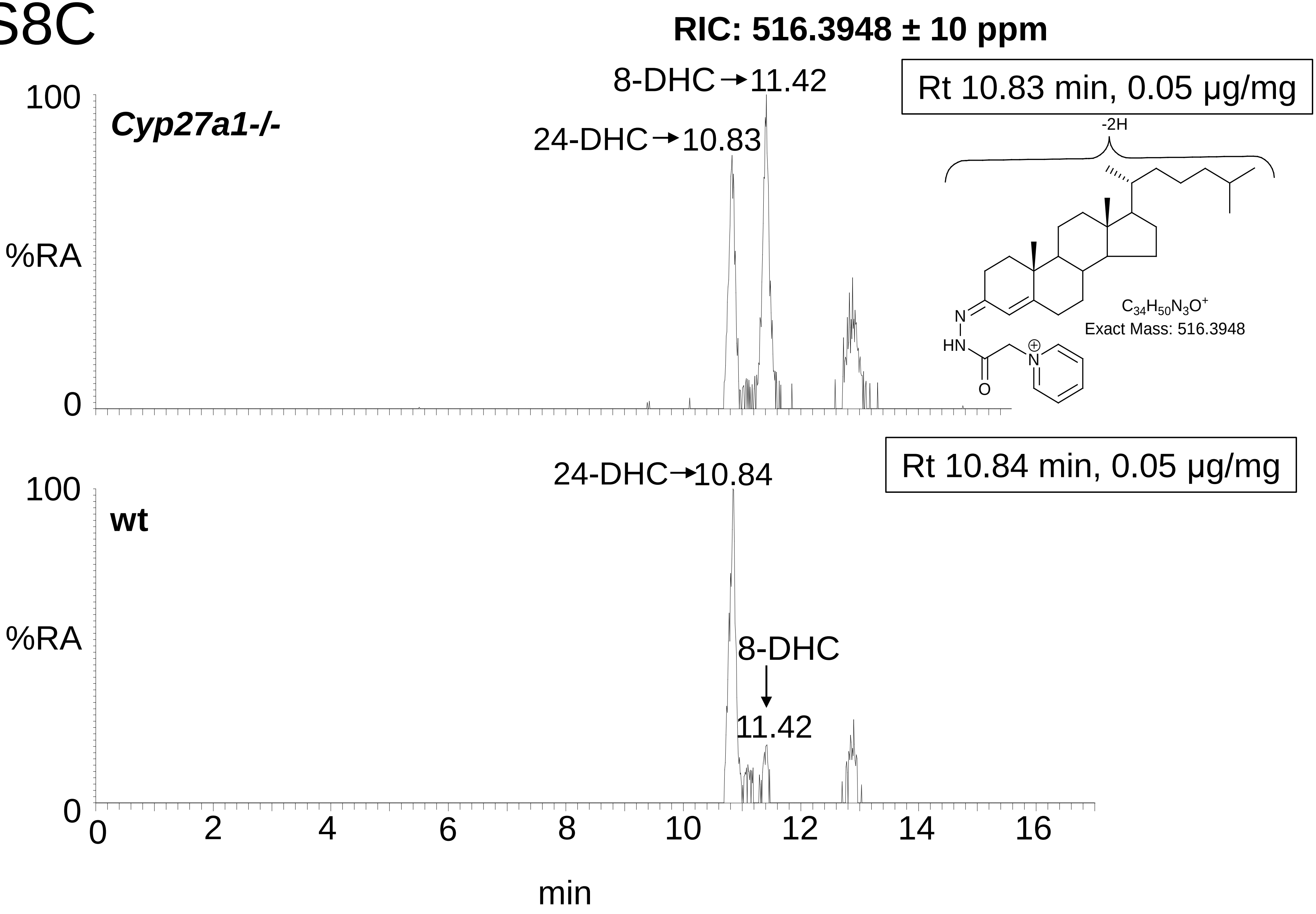

RIC: 516.3948 ± 10 ppm
Rt 10.83 min, 0.05 µg/mg
Cyp27a1-/-
8-DHC→11.42
24-DHC→10.83
100
%RA
0
-2H
N
HN
N
O
$C_{34}H_{50}N_3O^+$
Exact Mass: 516.3948
Rt 10.84 min, 0.05 µg/mg
wt
24-DHC→10.84
8-DHC
11.42
0
2
4
6
8
10
12
14
16
min

S8D

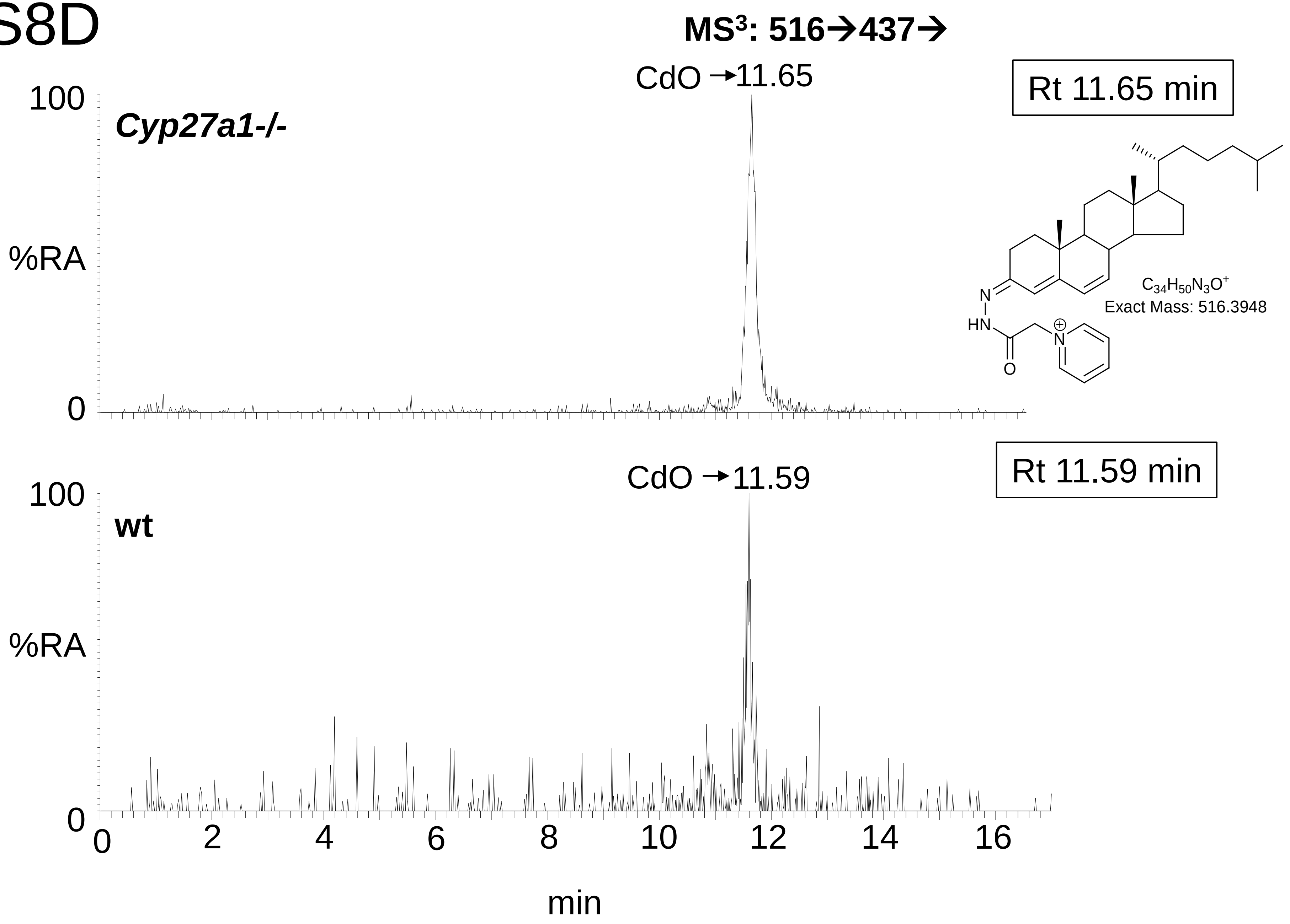

MS3: 516→437→
CdO →11.65
Rt 11.65 min
100
Cyp27a1-/-
%RA
0
C34H50N3O+
Exact Mass: 516.3948
N
HN
O
N
CdO →11.59
Rt 11.59 min
100
wt
%RA
0
0
2
4
6
8
10
12
14
16
min
